# Effects of exercise dosage on children with autism spectrum disorder: a systematic review and meta-analysis of randomized controlled trials

**DOI:** 10.3389/frcha.2025.1647280

**Published:** 2025-09-04

**Authors:** Baojian Hu, Qingxia Liang, Huiyi Jiang

**Affiliations:** ^1^ Department of Pediatrics, Children's Medical Center, The First Hospital of Jilin University, Lequn Branch, Changchun City, Jilin Province, China; ^2^ Enping People's Hospital, Enping City, Guangdong Province, China

**Keywords:** autism, exercise dosage, ACSM, meta-analysis, children

## Abstract

**Objective:**

To investigate the effects of exercise doses recommended by the American College of Sports Medicine (ACSM) on motor skills, social interaction, behavioral patterns, and verbal and non-verbal communication domains in children with autism.

**Methods:**

A systematic search was conducted across PubMed, Embase, Web of Science, and the Cochrane Library, focusing on the effects of physical activity on children with autism. Randomized controlled trials comparing exercise interventions with no intervention were included, and changes in motor skills, social interaction, behavioral patterns,and communication domains were assessed using standardized mean differences (SMD), 95% confidence intervals (CI), and p-values (*p* < 0.05).Interventions were categorized based on high or low adherence to exercise prescriptions developed or recommended by ACSM.Studies in which ≥70% of components met ACSM criteria were classified as having high adherence, while those with <70% were classified as having low adherence, based on thresholds established in previous literature. A fixed-effects or random-effects model was applied for meta-analysis, and subgroup comparisons were conducted.

**Results:**

A total of 27 studies (29 exercise interventions) involving 1,012 participants were included. In the motor skills domain,the pooled standardized mean difference (SMD) was 1.35, 95% confidence interval (CI) [0.66,2.03]. Subgroup analysis revealed that the high-adherence group showed an SMD of 1.44, 95% CI [0.51,2.36], while the low-adherence group showed an SMD of 1.26, 95% CI [0.15,2.36]. For the social interaction domain,the overall SMD was −0.22, 95% CI [−0.54,0.99]. The high-adherence subgroup had an SMD of −0.41, 95% CI [−0.62,−0.21], whereas the low-adherence group had an SMD of 0.42, 95% CI [−0.50,1.33]. In the behavioral patterns domain, the overall SMD was −0.79, 95% CI [−1.26,−0.32]. Subgroup analysis indicated an SMD of −0.42, 95% CI [−0.73,−0.11] for the high-adherence group and −2.79, 95% CI [−5.63,0.06] for the low-adherence group.For the verbal and non-verbal communication domain, the overall SMD was 0.33, 95% CI [−0.31,0.97]. Subgroup SMD were 0.21,95% CI [−0.14,0.57] for the high-adherence group and 0.59, 95% CI [−1.67,2.84] for the low-adherence group.

**Conclusion:**

Exercise interventions had a significant positive impact on motor skills and behavioral patterns in children with autism spectrum disorder (ASD). Interventions with high adherence to ACSM-recommended exercise dosages were more effective in improving motor skills, social interaction,and behavioral patterns compared to low-adherence dosages.Future evidence-based exercise prescriptions may be established for children with ASD, optimizing motor-functional outcomes.

**Systematic Review Registration:**

PROSPERO, identifier (CRD42024565241).

## 1 Introduction

ASD is a common neurodevelopmental disorder characterized by language and behavioral difficulties, including impaired social interaction,repetitive behaviors,communication deficits,and restricted interests and activities that manifest in early childhood and persist into adulthood.According to the U.S. Centers for Disease Control and Prevention (CDC) report released in 2025, the prevalence of autism among 8-year-old children reached 3.2% (equivalent to 1 in 31 children). This represents a significant increase compared to the rate of 1 in 150 children reported in 2000 (1). In China,more than 10 million individuals may be affected by autism, with over 2 million children aged 0 to 14.Males are four times more likely to be diagnosed with ASD than females.

In recent years, exercise interventions have gained increasing attention as a strategy to alleviate symptoms of autism,as physical activity is beneficial to physical, psychological,and emotional health—especially in children.Several studies have demonstrated that exercise interventions can improve motor skills,s ocial interaction,behavioral patterns, and both verbal and non-verbal communication in children and adolescents with autism. For instance, Roza et al. (2) reported that Tai Chi exercises reduced stereotypical behaviors in children with autism. However, systematic research in this area remains limited and is often based on small sample sizes, with limited investigation into population-level and individualized exercise dosage. For example, Felzer-Kim et al. (3) randomized trial revealed that a 10-week FMS intervention (4 weekly sessions ×15 min) did not significantly enhance motor proficiency in children with ASD compared to controls.

This review applied clearly defined exercise standards based on the guidelines of the American College of Sports Medicine (ACSM), which offer detailed recommendations for cardiorespiratory, resistance, and flexibility training in healthy children. However, it remains unclear whether interventions adhering closely to ACSM recommendations yield greater benefits for individuals with ASD than those with lower adherence. The objective of this systematic review is to compare the effects of high versus low adherence to ACSM-recommended exercise prescriptions on outcomes in individuals with ASD.

## 2 Methods

### 2.1 Literature search strategy

#### 2.1.1 Primary sources of literature

A comprehensive search was conducted in PubMed, Embase, Web of Science, and the Cochrane Library. The search covered all records from the inception of each database to February 1, 2025. In addition, manual searches were performed on relevant reviews and the reference lists of the included studies.

#### 2.1.2 Search terms and strategy

The search terms included the following Medical Subject Headings (MeSH) and keywords, used across PubMed, Embase, Web of Science, and the Cochrane Library: (“Pervasive Developmental Disorders”, “autism”, “ASD/Autism Spectrum Disorder”, “Asperger”, or“PDD-NOS”) and (“Exercise”or“Exercises” or “Sports” or “Physical Activity” or “Motor Activity” or “Training” or “endurance training” or“TaiChi”or“yoga”or “Balance”or“Resistance”or “Flexibility” or“Cardiovascular”or “Aerobic”).

The complete search strategy is presented in the Supplementary Material Data Sheet 1.

### 2.2 Inclusion criteria

Studies were included if they met the following criteria:

(a) Participants were children diagnosed with ASD;
(b) The intervention involved any form of exercise program, including resistance training, aerobic activity, or flexibility exercises;
(c) The behavioral outcomes of the intervention were quantitatively measured and could be expressed as percentage changes reflecting relative behavioral improvements.

### 2.3 Exclusion criteria

Studies were excluded if they met any of the following criteria:

(a) Reports, conference proceedings, narrative reviews, or editorials;
(b) Studies that did not include a comparison between exercise and non-exercise groups;
(c) Studies involving participants with disorders related to, but distinct from ASD;
(d) Studies in which participants concurrently received specific pharmacological treatments during the exercise intervention period;
(e) Duplicate data from multiple publications based on the same study;
(f) Studies in which the control intervention involved any form of treatment unrelated to exercise.

### 2.4 Study selection

All retrieved references were imported into EndNote 20, and duplicates were removed. Two reviewers independently screened the titles and abstracts to exclude irrelevant studies. Full texts of potentially relevant studies were retrieved and assessed for eligibility based on the inclusion and exclusion criteria. If either reviewer deemed a study potentially eligible, the full text was reviewed. Disagreements between reviewers regarding eligibility were resolved through discussion or adjudication by a third reviewer. There were no restrictions on participants' sex, body mass index (BMI), publication date, or language.

### 2.5 Data extraction

Data extraction was independently performed by two reviewers. Motor skills, social interaction, behavioral patterns, and both verbal and non-verbal communication were considered primary outcomes. A pre-designed Excel spreadsheet was used to extract relevant data, including publication characteristics (author names, country, year of publication), methodological characteristics (number of groups, group design, intervention measures, sample size), participant characteristics (age, sex ratio), exercise characteristics (intervention frequency, exercise intensity, duration, number of repetitions, and sets), and outcome characteristics.

When post-intervention outcome data were not explicitly reported in the text but presented in graphical form, data were extracted using Engauge Digitizer software. For studies with multiple follow-up assessments, only data obtained immediately after the intervention were extracted.

Following data extraction, exercise interventions were evaluated in terms of dosage and adherence. The dosage of each intervention was assessed based on the American College of Sports Medicine (ACSM) guidelines for the development and maintenance of cardiorespiratory, muscular, skeletal, and neuromotor functions in healthy children (see Table 1). Two reviewers independently assessed the adherence of each intervention to ACSM recommendations, considering dimensions such as frequency, intensity, and duration. Adherence levels were categorized based on predefined criteria.

**Table 1:** ACSM recommendations for cardiopulmonary exercise, resistance exercise, and stretching exercise in healthy children.

Exercise dosage	Cardiopulmonary exercise	Resistance exercise	Stretching exercises
Frequent occurrence	4–5/d.wk	1–2/d.wk (Non-consecutive days), Gradually increase to 2–3 days per week	5–7/d.wk
Intensity	Moderate intensity, 40%–59% VO_2_R/HRR, CR-10 scale rating of 3–4	Adjust the resistance, from medium to high intensity.	Stretching exercises until you feel the muscles are stretched tight or there is a slight discomfort.
Duration	20–30 min (up to 45–60 min)	Start with a set of 8–12 repetitions, and after about two weeks, increase to two sets. Each exercise should not exceed 8–10 repetitions.	Stretch for 10–30 s, repeat the stretch 2–4 times.

d.wk, (day/week); VO_2_R/HRR, reserve oxygen uptake or reserve heart rate; CR-10, (Borg CR-10 Grading Scale).

Each exercise component was scored on a scale from 0 to 2. A score of 2 indicated full compliance with ACSM standards, 1 indicated uncertainty, and 0 indicated non-compliance. Discrepancies between the two reviewers were resolved through discussion with a third reviewer until consensus was reached. Based on this scoring system, the proportion of components meeting ACSM-recommended exercise dosage was calculated for each study. Studies in which ≥70% of components met ACSM criteria were classified as having high adherence, while those with <70% were classified as having low adherence, based on thresholds established in previous literature.

Results with 95% confidence intervals (CIs) that do not include zero were considered statistically more reliable. Larger absolute values of the standardized mean difference (SMD) indicate greater between-group differences and may suggest greater clinical or practical significance. However, multiple factors must be carefully considered when interpreting these results to ensure the accuracy and reliability of the conclusions.

### 2.6 Statistical analysis

Meta-analyses were conducted using Review Manager (RevMan) version 5.4.1 to compare the outcomes across included studies. Studies were categorized into two subgroups representing high and low adherence to ACSM guidelines. Heterogeneity within each subgroup was assessed using Higgins' *I*^2^ statistic and interpreted based on the recommendations of the Cochrane Handbook. Heterogeneity was classified as low (*I*^2^ ≤ 40%), moderate (30% < *I*^2^ ≤ 60%), substantial (50% < *I*^2^ ≤ 90%), or considerable (*I*^2^ > 75%).

A fixed-effects model was applied when *I*^2^ ≤ 50%; otherwise, a random-effects model was used. Effect sizes were reported as standardized mean differences (SMD), where larger absolute SMD values indicate more pronounced effects, accompanied by 95% confidence intervals (CIs). When substantial heterogeneity was detected, meta-regression was performed to explore potential sources of variation. Publication bias was assessed using Begg's rank correlation test and Egger's linear regression test. A *p*-value < 0.05 was considered statistically significant. Sensitivity analyses were conducted by sequentially removing individual studies to examine the robustness of the results.

### 2.7 Risk of bias assessment

The methodological quality of the included studies was independently assessed by two reviewers using the quality appraisal criteria recommended by the Cochrane Collaboration. All studies included in this review were randomized controlled trials (RCTs). According to the Cochrane Handbook, the revised Cochrane risk of bias tool for randomized trials (RoB 2) was employed. RoB 2 provides a structured framework for evaluating the risk of bias in individual outcomes of randomized trials.The tool evaluates several domains, including random sequence generation, allocation concealment, blinding of participants and personnel, blinding of outcome assessment, incomplete outcome data, selective reporting, and other potential sources of bias. Reviewers rated each study according to the Cochrane Handbook guidelines. Each domain was rated as “low risk”, “some concerns” or “high risk” of bias. An overall risk of bias judgment was made as follows: “low risk” if all domains were rated as low risk; “some concerns” if at least one domain raised concerns but none were rated as high risk; and “high risk” if one or more domains were rated as high risk.

## 3 Results

### 3.1 Study selection

A total of 12,392 records were retrieved from four databases: PubMed, Web of Science, Embase, and the Cochrane Library. After removing 2,352 duplicates, 10,040 articles remained. Following title and abstract screening, 9,876 records were excluded, leaving 164 full-text articles for eligibility assessment. Of these, 137 articles were excluded due to reasons such as non-randomized study design or lack of relevant outcome data. Ultimately, 27 studies were included in this review. Two of these studies [Ansari et al. (4); Li et al. (5)] contained two eligible exercise interventions, yielding a total of 29 included comparisons.

**Figure 1:**
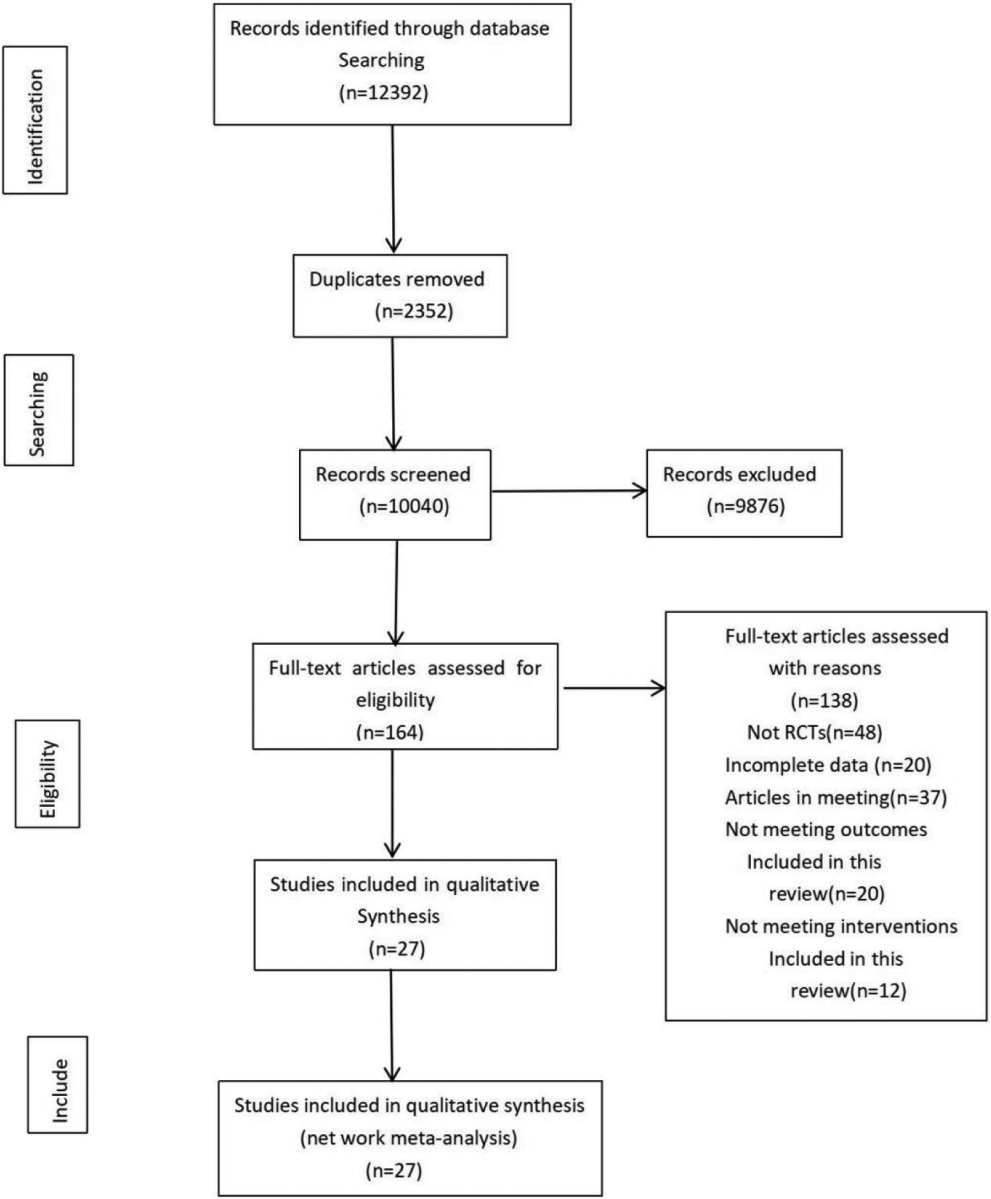
Literature search and screening flowchart.

The included studies were published between 2010 and 2024 and involved authors such as: Ansari et al. (4), Li et al. (5), Manizheh (6), Jin (7), Piedad et al. (8), Mahrokh et al. (9), Felzer-Kim and Hauck (3), Amir et al. (10), Xingda et al. (11), Ketcheson et al. (12), Yumi et al. (13), Li (14), Vieira et al. (15), Pan (16, 17), Yamaner et al. (18), Zhao et al. (19), Kelong et al. (20), Koenig et al. (21), Toscano et al. (22), Wang et al. (23), Xu et al. (24), Zhou et al. (25), Sotoodeh et al. (26), Choi et al. (27), Roza et al. (2), and Ezginur et al. (28).

(See Figure 1 for the PRISMA flow diagram).

### 3.2 Characteristics and quality assessment of included studies

#### 3.2.1 Basic characteristics of included studies

A total of 27 randomized controlled trials were included, comprising 1,012 participants—551 in the intervention groups and 461 in the control groups. Gender distribution was explicitly reported in 22 studies, with a reported male-to-female ratio of 575:139. Four studies included only male participants. Two studies involved more than one exercise intervention group.

Among the included studies, 10 were conducted in China, 6 in Iran, 4 in the United States, 2 each in South Korea and Turkey, and 1 each in Colombia, Brazil, and Portugal. Participants were primarily recruited through schools and rehabilitation institutions (see Table 2).

**Table 2:** Incorporating literature feature descriptions.

Author	Country	Year	Experimental group age (mean ± standard deviation) Control group age (mean ± standard deviation)	(Experimental group total)/Male/Female (Control group total)/Male/Female	Experimental group intervention measures	Result
Ansari et al.	Iran	2020	EG(Swimming): 10.6 (2.5) EG(Karate):10.8 (2.14) CG:10.8 (2.44)	EG((Swimming):10/10/0 EG((Karate):10/10/0 CG:10/10/0	Swimming-based exercise, karate Intervention duration: 10 weeks Frequency: Twice a week Duration: 60 min	Motor skills Zone
Manizheh	Iran	2019	EG:8.4 (2.01) CG:8.44 (1.94)	EG:15/11/4 CG:15/12/3	Regular exercise Intervention duration: 10 weeks Frequency: 3 times per week Duration: 60 min	Motor skills Zone Behavioral pattern recognition Zone Language and non-verbal communication ability Zone
Jin	China	2024	EG:8.35 (1.75) CG:7.27 (1.35)	EG:9/7/2 CG:17/14/3	CPRT Intervention duration: 9 weeks Frequency: 3 times per week Duration: 80 min	Motor skills Zone
Piedad et al	Colombia	2023	EG:5.8 (1.39) CG:5.8 (1.61)	EG:10/8/2 CG:10/9/1	Structured physical exercise Intervention duration: 8 weeks Frequency: 3 times per week Duration: 60 min	Motor skills Zone
Mahrokh et al.	Iran	2023	EG:9.2 (0.6) CG:9.4 (0.5)	EG:12/12/0 CG:12/12/0	Multimodal Exercise Intervention Duration: 8 weeks Frequency: 3 times per week Duration: 45 min	Motor skills Zone
Felzer-Kim et al.	United States	2020	EG:4.5 (0.58) CG:4.5 (0.59)	EG:8/7/1 CG:6/3/3	Basic Motor Skills Intervention Duration: 10 weeks Frequency: 4 times per week Duration: 15 min	Motor skills Zone
Amir et al.	Iran	2023	EG:9.00 (1.31) CG:8.13 (1.36)	EG:8/5/3 CG:8/4/4	Comprehensive Physical Training Intervention Duration: 8 weeks Frequency: 3 times per week Duration: 30 min	Motor Skills Area Social Interaction Zone Behavioral Patterns Zone Language and Non-verbal Communication Zone
Xingda et al.	China	2024	EG:11.11 (2.52) CG:12.75 (2.31)	EG:9/6/3 CG:8/6/2	Yoga Intervention Duration: 8 weeks Frequency: 3 times per week Duration: 45–50 min	Motor Skills Area Social Interaction Zone Behavioral Patterns Zone Language and Non-verbal Communicatio Zone
Ketcheson et al.	United States	2017	EG:4.87 (0.61) CG:5.05 (0.61)	EG:11/9/2 CG:9/6/3	CPRT Intervention duration: 8 weeks Frequency: 5 times per week Duration: 240 min	Motor skills Zone
Yamaner et al.	United States	2016	EG:10.25 (2.38) CG:10.00 (2.83)	EG:8/8/0 CG:6/5/1	Taekwondo Intervention Duration: 8 weeks Frequency: Twice a week Duration: 50 min	Motor skills Zone
Li	South Korea	2019	EG:14.6 (3.0) CG:13.2 (3.4)	EG:16/NR/NR CG:18/NR/NR	Special Exercise Program Intervention Duration: 12 weeks Frequency: 3 times per week Duration: 60 min	Motor skills Zone Language and non-verbal communication Zone
Vieira et al.	Portugal	2016	EG:NR CG:NR	EG:6/NR/NR CG:11/NR/NR	Special Exercise Program Intervention Duration: 12 weeks Frequency: 3 times per week Duration: 60 min	Motor skills Zone
Pan	China	2017	EG:9.68 (1.61) CG:8.49 (1.76)	EG:11/11/0 CG:11/11/0	Physical Activity Intervention Duration: 12 weeks Frequency: Twice a week Duration: 70 min	Motor skills Zone
Yamaner et al.	Turkey	2022	EG:NR CG:NR	EG:15/NR/NR CG:15/NR/NR	Sports activities Intervention duration: 12 weeks Frequency: Twice a week Duration: 70 min	Motor skills Zone
Zhao et al.	China	2021	EG:6.68 (2.43) CG:7.31 (4.26)	EG:17/13/4 CG:8/6/2	Graded Comprehensive Practice Intervention Duration: 5 weeks Frequency: 3 times per week Duration: 60 min	Motor Skills Area Social Interaction Zone Language and Non-Verbal Communication Zone
Kelong et al.	China	2020	EG:5.13 (0.61) CG:4.68 (0.72)	EG:15/12/3 CG:14/13/1	Mini Basketball Training Intervention Duration: 12 weeks Frequency: 5 times per week Duration: 40 min	Social interaction Zone
Koenig et al.	United States	2012	T:9.58(NR) C:8.58(NR)	T:24/19/5 C:22/18/4	Yoga Intervention Duration: 16 weeks Frequency: 7 times per week Duration: 10–15 min	Social Interaction Zone Behavioral Patterns Zone Language and Non-verbal Communication Zone
Li et al.	South Korea	2019	EG(Aquatic sports):10.22 (0.44) EG(general sports):10.34 (0.39) CG:10.29 (0.35)	EG(Aquatic sports):10/N/N EG(general sports):10/N/N CG:9/N/N	Aquatic sports, general sports Intervention duration: 16 weeks Frequency: Once a week Duration: 50 min	Social Interaction Zone Behavioral Pattern Zone
Toscano et al.	Brazil	2022	EG:7.9 (3.3) CG:8.1 (3.2)	EG:127/N/N CG:62/N/N	Physical Exercise Intervention Duration: 48 weeks Frequency: Twice a week Duration: 30 min	Social Interaction Zone Behavioral Patterns Zone Language and Non-verbal Communication Zone
Wang et al	China	2020	EG:5.11 (0.65) CG:4.7 (0.7)	EG:18/15/3 CG:15/13/2	MBTP Intervention duration: 12 weeksFrequency: 5 times per week Duration: 40 min	Social Interaction Zone Behavioral Pattern Zone
Xu et al.	China	2018	EG:14.8 (6.1) CG:15.5 (5.1)	EG:52/40/12 CG:54/42/12	General sports activities Intervention duration: 16 weeks Frequency: 3 times per week Duration: 90 min	Social Interaction Zone Behavioral Pattern Zone
Dongyue Zhou	China	2023	EG:6.4 (2.07) CG:5.94 (1.79)	EG:32/28/4 CG:26/22/4	MBTP Intervention duration: 12 weeks Frequency: 5 times per week Duration: 40 min	Social Interaction Zone Behavioral Pattern Zone
Zhou et al.	Iran	2017	EG:10.8 (2.36) CG:10.5 (1.87)	EG:15/11/4 CG:14/10/4	Yoga Intervention duration: 8 weeks Frequency: 3 times per week Duration: 30 min	Social Interaction Zone Verbal and Non-verbal Communication Zone
Choi et al.	China	2022	EG:10.86 (1.99) CG:11.17 (1.93)	EG:23/21/2 CG:32/26/6	Morning jogging Intervention duration: 12 weeks Frequency: 3 times per week Duration: 30 min	Behavioral Pattern Zone
Roza et al.	Iran	2021	EG:9.6 (1.4) CG:9.6 (1.4)	EG:12/10/2 CG:11/9/2	Tai Chi Intervention Duration: 12 weeks Frequency: 3 times per week Duration: 45 min	Behavioral Pattern Zone
Ezginur et al.	Turkey	2024	EG:12.33 (1.58) CG:12.57 (1.52)	EG:30/14/16 CG:30/20/10	Sports activities Intervention duration: 12 weeks Frequency: Twice a week Duration: 90 min	Verbal and Non-verbal Communication Zone
Pan	China	2010	EG:7.27 (1.25) CG:7.20 (0.89)	EG:8/8/0 CG:8/8/0	Aquatic Sports Swimming Intervention Duration: 10 weeks Frequency: Twice a week Duration: 90 min	Social Interaction Zone Verbal and Non-verbal Communication Zone

EG, Experimental group; CG, Control group.

The duration of interventions ranged from 5 to 48 weeks, with exercise frequency varying between 1 and 5 days per week. All studies implemented exercise interventions, either supervised or home-based. Intervention types primarily included aerobic exercise, yoga, Tai Chi, swimming, karate, and mini-basketball.

Mean and standard deviation (SD) values were recorded for post-intervention outcomes in both intervention and control groups across the following domains: motor skills (Table 3), social interaction (Table 4), behavioral patterns (Table 5), and verbal/non-verbal communication (Table 6). After classifying the interventions, 28 studies involved cardiorespiratory exercise, 20 included resistance training, and 7 included flexibility exercises. Twenty-two studies implemented multi-modal exercise programs (Table 7).

**Table 3:** Motor skill area group-related literature and research intervention outcomes.

First author	Year of Publication	Evaluation Scale	Mean value of the experimental group after intervention	Standard deviation of the experimental group after intervention	Mean value of the control group after intervention	Standard deviation of the control group after intervention
Amir et al	2020	Stork Standing Test (Modified Version)	9.6	1.34	6.7	1.59
Amir et al. (2)	2020	Stork Standing Test (Modified Version)	13	1.49	6.7	1.59
Manizheh	2019	Gross Motor Development Test-2 Scale	47	5.05	34.44	8.22
Jin	2024	Gross Motor Development Test-3 Scale	22.0792	2.9703	17.62398	2.6732
Piedad et al	2023	Simple Developmental Scale-3	50.6	7.56	42.9	6.32
Mahrokh Dehghani	2023	Walking Test Experiment	5.18	0.82	6.32	1.66
Felzer-Kim et al.	2020	Gross Motor Development Test-3 Scale	20.75	16.45	12.4	7.7
Amir et al.	2023	Sargent jump test	3.1	1.1	1.6	0.59
Xingda et al.	2024	Motor Assessment Scale-2	9.33	3.71	4.5	0.93
Ketcheson et al.	2017	Gross Motor Development Test-2 Scale (Revised Version)	34.63	7.66	16.3	7.66
Yumi et al	2016	Balance test	8.26	4.09	11.47	1.31
Li	2019	Visual-Motor Integration Ability Test	73.463	23.757	51.04	23.755
Vieira et al.	2016	Bunny Movement Proficiency Test-2 (Revised Edition)	35.17	17.75	30.27	7.55
Pan	2017	Bunny Movement Proficiency Test-2	60.09	10.11	49.82	5.85
Yamaner et al.	2022	Reaction test	7.94	0.079	6.97	0.079
Zhou et al.	2021	Autism Behavior Checklist	3.89	4.78	5.13	5.87

**Table 4:** Social interaction block-related literature and research intervention outcomes.

First author	Year of publication	Evaluation scale	Mean value of the experimental group after intervention	Standard deviation of the experimental group after intervention	Mean value of the control group after intervention	Standard deviation of the control group after intervention
Kelong et al.	2020	Social Response Scale	85.27	29.41	97.14	22.14
Amir et al.	2023	Autism Rating Scale-2	13	11.98	15.75	10.29
Xingda et al.	2024	Abnormal Behavior Checklist	20.89	4.54	26.63	2.39
Li et al.	2019	Communication Execution Rate Scale	32.89	11.28	19.87	9.78
Li et al. (2)	2019	Communication Execution Rate Scale	30.17	10.15	19.87	9.78
Toscano et al.	2022	Autism Trait Assessment Scale	3.3	4.2548	5.11	4.1178
Wang et al.	2020	Social Response Scale-2	82.22	27.55	95.93	19.47
Xu et al.	2018	Autism Treatment Evaluation Checklist	14.7	6.7	19.2	6.9
Zhao et al.	2021	Autism Behavior Checklist	13	10.19	17.25	8.14
Zhou et al.	2023	Abnormal Behavior Checklist	20.0073	1.7359	27.1845	2.1498
Koenig et al.	2012	Abnormal Behavior Checklist (Community)	10.88	2.905	9.55	2.905
Sotoodeh et al.	2017	Autism Treatment Evaluation Checklist	9.92	10.744	10.98	4.2433
Pan	2010	Social Behavior Scale	45.75	5.44	51.75	5.38

**Table 5:** Behavioral patterns in district groups: relevant literature and research intervention outcomes.

First author	Year of publication	Evaluation scale	Mean value of the experimental group after intervention	Standard deviation of the experimental group after intervention	Mean value of the control group after intervention	Standard deviation of the control group after intervention
Manizheh	2019	Autism Diagnostic Observation Schedule	76.2	11.62	67.84	25.99
Amir et al.	2023	Autism Rating Scale-2	11.75	9.36	14.37	7.71
Xingda et al	2024	Abnormal Behavior Checklist	13.78	3.53	16.63	2.62
Li et al.	2019	Repetitive Behavior Observation Scale	159.12	123.57	875.88	199.97
Li et al. (2)	2019	Repetitive Behavior Observation Scale	170.57	134.34	875.88	199.97
Choi et al.	2022	Abnormal Behavior Checklist	15.33	8.73	18.24	8.1
Wang et al.	2020	Repetitive Behavior Scale-Revised	11.22	9.31	23.13	14.19
Zhou et al.	2023	Repetitive Behavior Scale	1.38997	0.42008	1.78092	0.51073
Roza et al.	2021	Autism Rating Scale-2	8.8333	5.37	12.08	6.097
Koenig et al.	2012	Abnormal Behavior Checklist (Community)	4.33	0.48	4.55	0.48
Toscano et al.	2022	Autism Trait Assessment Scale	4	5.23	4.76	4.5

**Table 6:** Verbal and nonverbal communication ability group-related literature and research intervention outcomes.

First author	Year of publication	Evaluation scale	Mean value of the experimental group after intervention	Standard deviation of the experimental group after intervention	Mean value of the control group after intervention	Standard deviation of the control group after intervention
Amir et al.	2023	Autism Rating Scale-2	16.37	14.62	15.37	12.65
Ezginur et al.	2024	Social Skills Assessment Form	2.4	0.27	1.67	0.16
Pan	2010	Social Behavior Scale	46.25	3.99	43.88	3.48
Xingda et al.	2024	Abnormal Behavior Checklist	5.56	1.42	6.25	1.67
Li	2019	Childhood Autism Rating Scale	−6.5	5.4	−9.8	7.9
Xu et al.	2018	Autism Treatment Evaluation Checklist	12.2	3.5	16.2	3.8
Zhao et al.	2021	Autism Behavior Checklist	10.11	7.46	8.63	6.8
Koenig et al	2012	Abnormal Behavior Checklist (Community)	1.96	0.117	2	0.117
Sotoodeh et al.	2017	Autism Treatment Evaluation Checklist	13.9807	7.494	13.8264	4.55
Toscano et al.	2022	Autism Trait Assessment Scale	4.397	2.789	4.75	2.19
Manizheh	2019	Autism Diagnostic Observation Schedule	13	4.42	8.77	2.33

**Table 7:** Exercise intervention scores assessed according to the recommendations of the American college of sports medicine (ACSM).

Author, year		Cardiopulmonary exercise						Resistance exercise								Stretching exercises						Percentage
		Frequent occurrence		Intensity		Duration		Frequent occurrence		Intensity		Repeat		Group		Frequent occurrence		Intensity		Duration		
		4–5 times/d.wk		Moderate intensity, 40%–59% VO2R/HRR, CR-10 scale rating of 3–4		20–30 min (Up to 45–60 min)		1–2/d.wk (Non- consecutive days), Gradually increase to 2–3 days per week		Adjust the resistance, from medium to high intensity.		8–12 times		1–2 group		5–7 Points/d.wk		Stretching exercises until you feel the muscles are stretched tight or there is a slight discomfort.		Stretch for 10–30 seconds, repeat the stretch 2–4 times.		
Ansari et al	2020	2 times	0 Points	Swimming	2 Points	60 Min	2 Points	2 days	2 Points	Aquatic exercises	2 Points	NR	1 Points	NR	1 Points							10/14 (71%)
Ansari et al. (2)	2020	2 times	0 Points	Karate	2 Points	60 Min	2 Points									2 times	0 Points	NR	1 Points	NR	1 Points	6/12 (50%)
Manizheh	2019	3 times	0 Points	Jogging, jumping	2 Points	60 Min	2 Points	3 days	2 Points	Throwing a ball, catching a ball	2 Points	NR	1 Points	NR	1 Points							10/14 (71%)
Jin	2024	3 times	0 Points	Run, jump	2 Points	80 Min	0 Points	3 days	2 Points	Throwing, striking	2 Points	NR	1 Points	NR	1 Points							8/14 (57.1%)
Piedad et al	2023	3 times	0 Points	Running, jumping	2 Points	60 Min	2 Points	3 days	2 Points	Pitching	2 Points	NR	1 Points	NR	1 Points	3 times	0 Points	Stretching	2 Points	2–3 times	2 Points	14/20 (70%)
Mahrokh et al.	2023	3 times	0 Points	Aerobic dance	2 Points	45 Min	2 Points									3 times	0 Points	Dynamic stretching	2 Points	NR	1 Points	7/12 (58.3%)
Felzer-Kim et al.	2020	4 times	2 Points	Run, jump	2 Points	15 Min	0 Points	4 days	0 Points	Throwing a ball, hitting a ball	2 Points	NR	1 Points	NR	1 Points							8/14 (57.1%)
Amir et al.	2023	3 times	0 Points	Rhythmic movements	2 Points	10–14 Min	0 Points	3 days	2 Points	Elastic Band Training	2 Points	8–10 times	2 Points	2 Groups	2 Points							10/14 (71%)
Xingda et al.	2024	3 times	0 Points	Yoga	2 Points	45–50 Min	2 Points	3 days	2 Points	Lifting legs, raising hands, etc.	2 Points	NR	1 Points	NR	1 Points	3 times	0 Points	Posture Stretching	2 Points	5 Min	2 Points	14/20 (70%)
Ketcheson et al.	2017							5 days	0 Points	Pitching	2 Points	NR	1 Points	NR	1 Points							4/8 (50%)
Yamaner et al.	2016	2 times	0 Points	Taekwondo	2 Points	50 Min	2 Points	2 days	2 Points	Push-ups.	2 Points	NR	1 Points	NR	1 Points							10/14 (71%)
Li	2019	3 times	0 Points	Running, four sets of crawling	2 Points	60 Min	2 Points	3 days	2 Points	Pitching	2 Points	NR	1 Points	NR	1 Points							10/14 (71%)
Vieira et al.	2016	1 times	0 Points	Trampoline training	2 Points	45 Min	2 Points	1 days	2 Points	Jumping	2 Points	NR	1 Points	NR	1 Points							10/14 (71%)
Pan	2017	2 times	0 Points	Table tennis, jogging	2 Points	70 Min	0 Points	2 days	2 Points	Pitching	2 Points	NR	1 Points	NR	1 Points							8/14 (57.1%)
Yamaner et al.	2022	3 times	0 Points	Aerobic exercise	2 Points	45 Min	2 Points	3 days	2 Points	Pitching	2 Points	NR	1 Points	NR	1 Points							10/14 (71%)
Zhao et al.	2021	3 times	0 Points	Running, jumping, and other sports training	2 Points	60 Min	2 Points	3 days	2 Points	Jumping, lifting legs	2 Points	NR	1 Points	NR	1 Points							10/14 (71%)
Kelong et al	2020	5 times	2 Points	Mini basketball	2 Points	40 Min	2 Points															6/6 (100%)
Koenig et al.	2012	7 times	0 Points	Yoga	2 Points	10–15 Min	0 Points	7 days	0 Points	Yoga	2 Points	NR	1 Points	NR	1 Points	7 times	2 Points	Stretching	2 Points	NR	1 Points	11/20 (55%)
Li et al.	2019	1 times	0 Points	Running	1 Points	50 Min	2 Points									1 times	0 Points	Water Stretching	1 Points	NR	1 Points	5/12 (41.7%)
Li et al. (2)	2019	1 times	0 Points	Basic movements	1 Points	50 Min	2 Points															3/6 (50%)
Toscano et al.	2022	2 times	0 Points	Physical exercise	2 Points	30 Min	2 Points	2 days	2 Points	Elastic bands, climbing	2 Points	NR	1 Points	NR	1 Points							10/14 (71%)
Wang et al	2020	5 times	2 Points	Mini basketball	2 Points	40 Min	2 Points															6/6 (100%)
Xu et al.	2018	3 times	0 Points	Rehabilitation based on exercise	1 Points	90 Min	0 Points															1/6 (16.7%)
Zhou et al.	2023	5 times	2 Points	Mini basketball	2 Points	40 Min	2 Points															6/6 (100%)
Sotoodeh et al.	2017	3 times	0 Points	Yoga	2 Points	30 Min	2 Points	2 days	2 Points	Yoga	2 Points	NR	1 Points	NR	1 Points	3 times	0 Points	Water Stretching	2 Points	5 Min	2 Points	14/20 (70%)
Choi et al.	2022	2 times	0 Points	Morning jog	2 Points	30 Min	2 Points	2 days	2 Points	NR	1 Points	NR	1 Points	1–2 Group	2 Points							10/14 (71%)
Roza et al	2021	3 times	0 Points	Tai Chi	2 Points	45 Min	2 Points	3 days	0 Points	Leg lift, etc.	2 Points	NR	1 Points	NR	1 Points							10/14 (71%)
Ezginur et al.	2024	2 times	0 Points	Sports activities	2 Points	90 Min	0 Points															2/6 (33.3%)
Pan	2010	2 times	0 Points	Aquatic sports	2 Points	60 Min	2 Points	2 days	2 Points	Warm-up in the water	2 Points	NR	1 Points	2 Groups	2 Points							11/14 (78.6%)

#### 3.2.2 Quality assessment of included studies

The methodological quality of the included studies was assessed using the risk of bias criteria recommended by the Cochrane Collaboration. All studies were judged to have a low risk of bias for random sequence generation. Among the 29 included exercise interventions, 23 were considered to have a low risk of bias for allocation concealment, while 6 studies did not report allocation methods and were rated as having an unclear risk.

Blinding of participants and personnel showed a relatively high risk of bias, as double-blinding was difficult to implement in exercise-based interventions. Outcome assessor blinding also exhibited elevated risk, as most outcomes were measured using subjective rating scales. Incomplete outcome data were observed in six cases due to missing behavioral scores, indicating a potential risk of bias.

**Figure 2:**
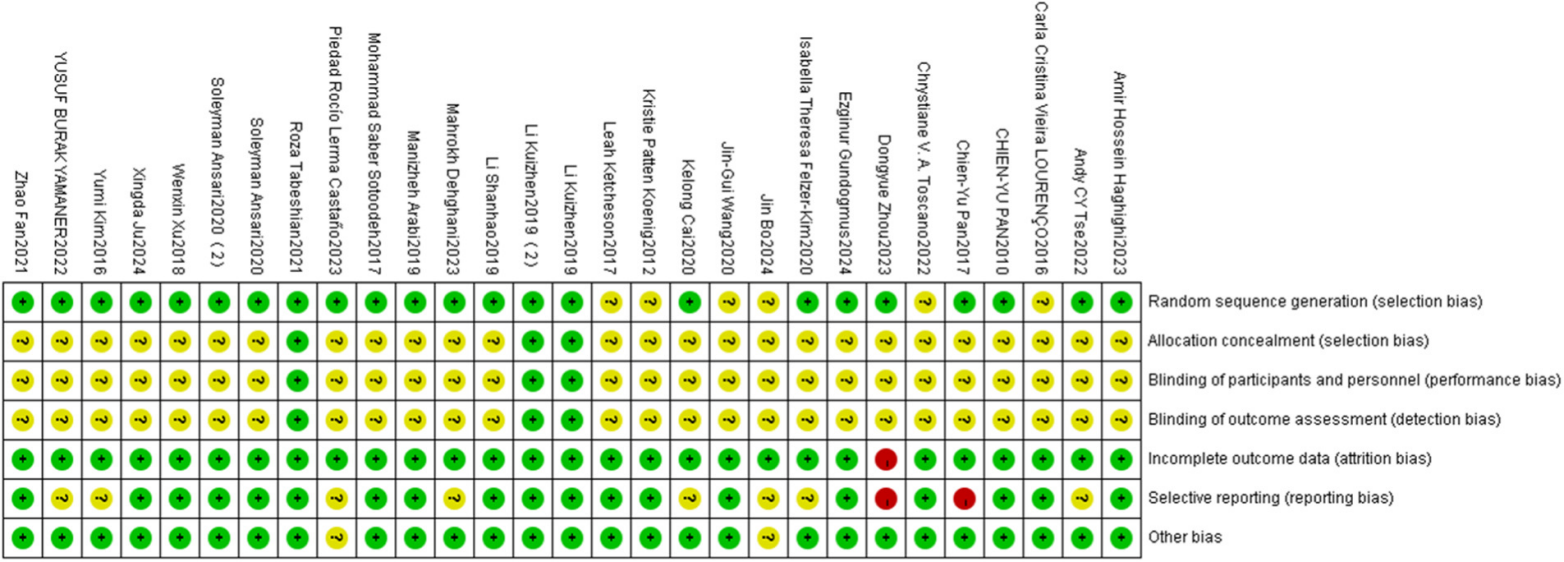
Risk of bias assessment for each included study.

Selective reporting was rated as low risk in 21 studies, moderate in 6 studies due to a lack of detailed explanations for participant dropout, and high in 2 studies. Additionally, two studies were identified as having other potential sources of bias (see Figure 2).

### 3.3 Effects of adherence to ACSM guidelines on motor skills, social interaction, behavioral patterns, and communication domains

Among the 27 included studies (comprising 29 exercise interventions), 18 met the threshold for high adherence to ACSM guidelines (≥70%), while 11 were categorized as low adherence (<70%). Low adherence was attributed partly to discrepancies between the reported exercise dosage and the ACSM recommendations, and partly to insufficient reporting, which limited accurate evaluation of the exercise prescription.

#### 3.3.1 Effects of adherence to ACSM guidelines on motor skills

When analyzing outcomes related to motor skills, 10 studies demonstrated high adherence to ACSM guidelines (≥70%), while 6 studies showed low adherence (<70%). Heterogeneity analysis revealed a high level of heterogeneity (*I*^2^ = 85%), prompting the use of a random-effects model for meta-analysis. The pooled standardized mean difference (SMD) for post-intervention motor skills between exercise and control groups was 1.35 (95% CI: [0.66,2.03], *p* < 0.001). These findings indicate a significant positive effect of exercise on motor skills in children with ASD.

Subgroup analysis yielded the following results: in the high adherence subgroup, the SMD was 1.44 (95% CI: [0.51,2.36], *p* = 0.002), with *I*^2^ = 86%, suggesting that high adherence to ACSM guidelines may be associated with greater improvements in motor skills. The confidence interval did not include zero, indicating statistical significance. However, the high heterogeneity indicates substantial variability across these studies.

In the low adherence subgroup, the SMD was 1.26 (95% CI: [0.15,2.36], *p* = 0.030), also with *I*^2^ = 86%. This result likewise suggests a positive effect of exercise on motor skills, as the confidence interval did not include zero. Nonetheless, the high heterogeneity suggests considerable differences in study design, sample characteristics, or intervention protocols, and thus the findings should be interpreted with caution.

**Figure 3:**
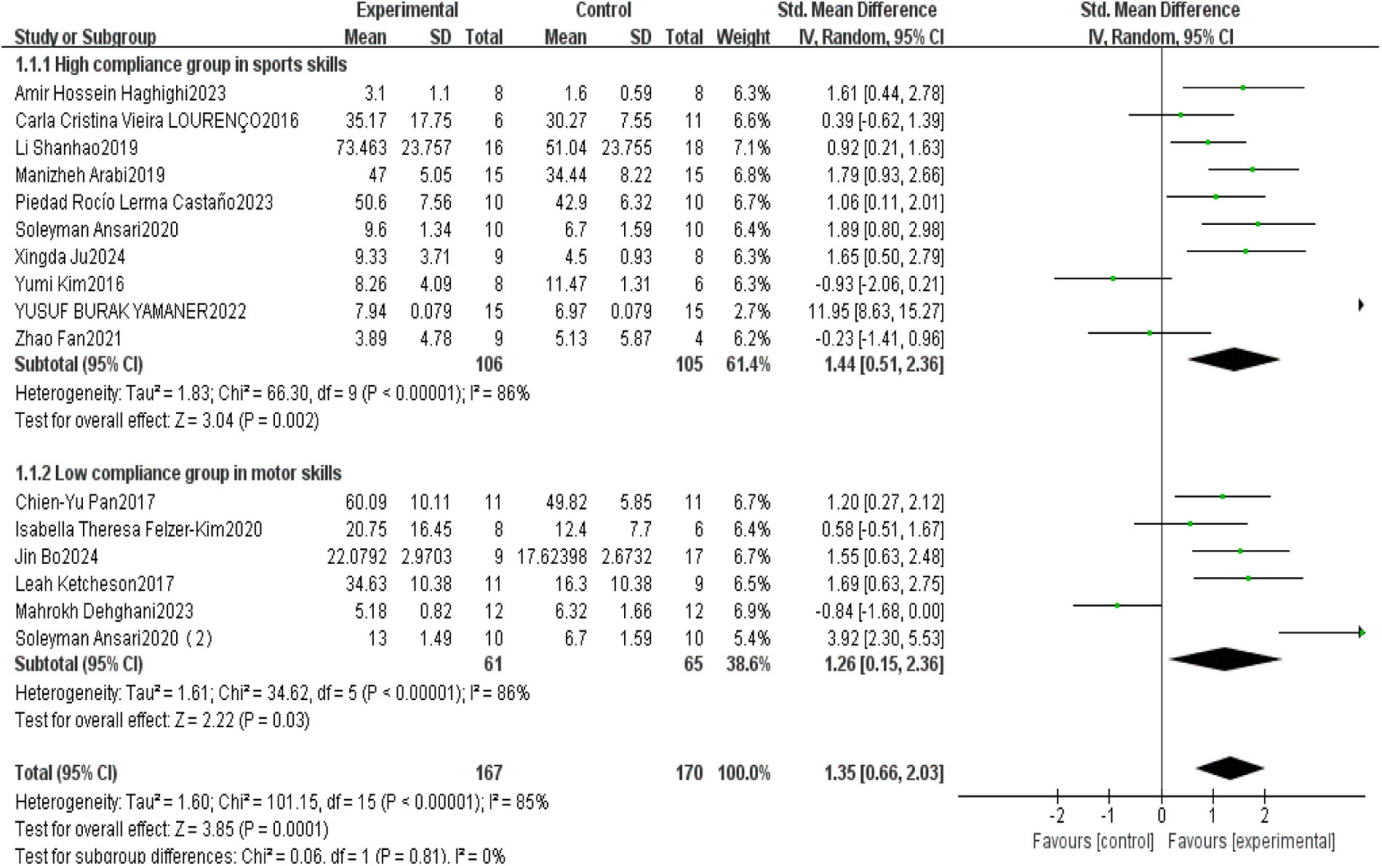
Forest plot of the impact of exercise intervention on motor skill domains.

Overall, exercise interventions with high adherence to ACSM guidelines were more strongly associated with improvements in motor skills among children with ASD compared to those with low adherence, though the substantial heterogeneity across studies warrants cautious interpretation (see Figure 3).

**Figure 4:**
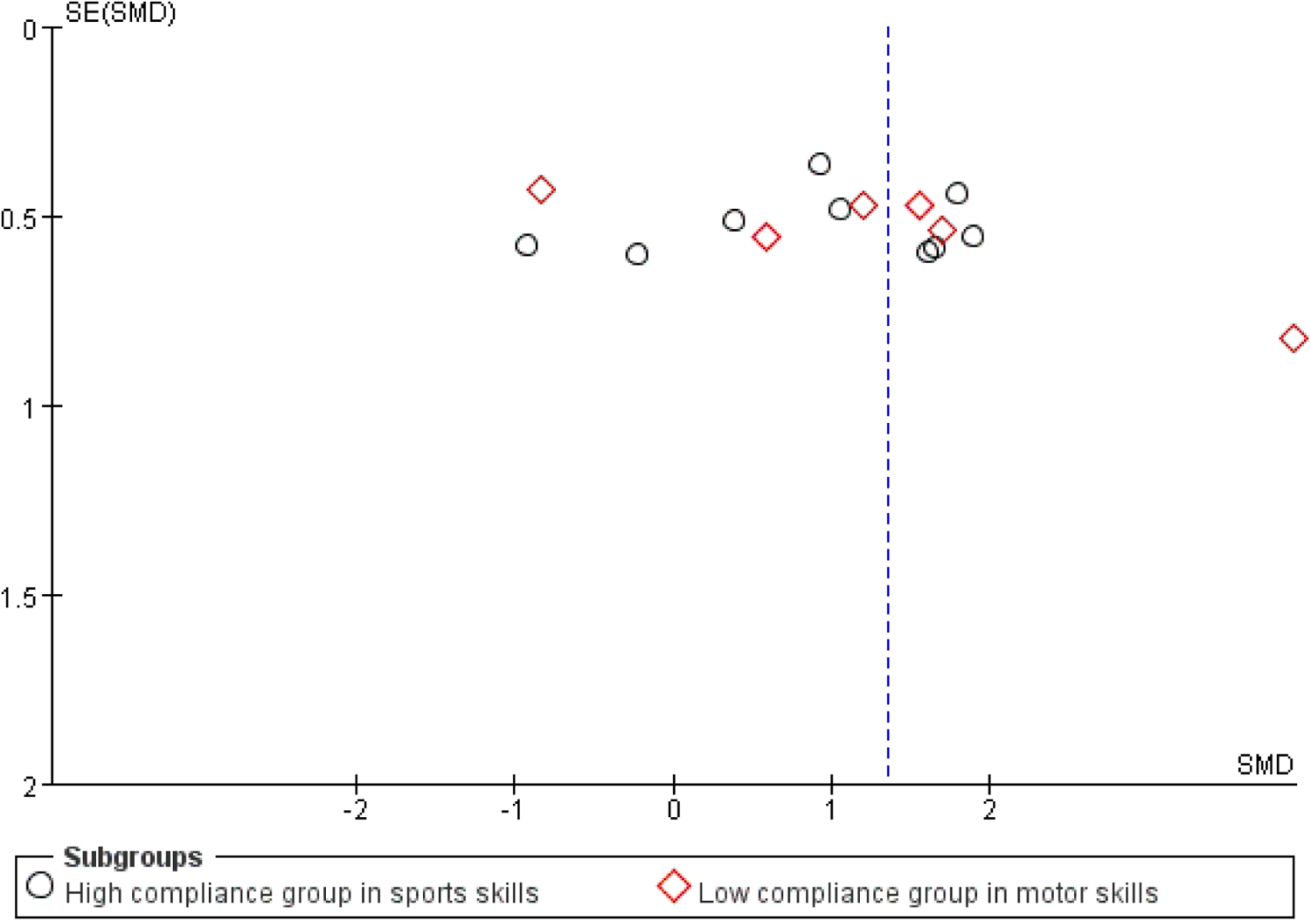
Funnel plot of the impact of exercise intervention on motor skill domains.

Publication bias and sensitivity analyses were conducted to assess the robustness of the findings. The funnel plot (Figure 4) appeared symmetrical, suggesting no apparent publication bias. Begg's test (*p* = 0.105) indicated non-significant bias, whereas Egger's test (*p* = 0.001) revealed significant publication bias—potentially due to small-sample studies tending to report larger effect sizes, while larger studies may be underrepresented if their results were not statistically significant.

**Figure 5:**
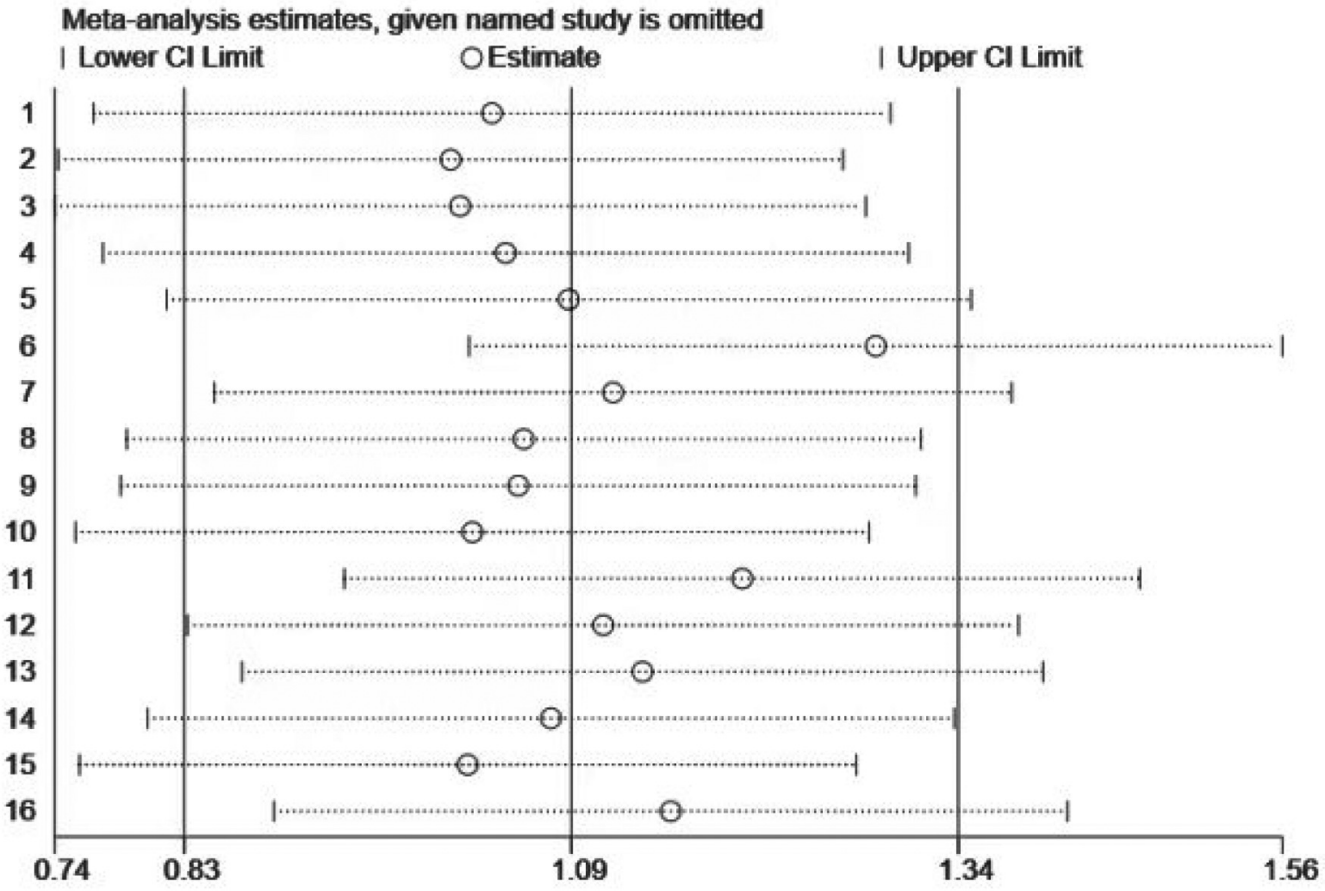
Sensitivity analysis of the impact of exercise intervention on motor skill domains.

Sensitivity analysis (Figure 5) showed that excluding any single study did not materially alter the overall effect size, indicating that the results were stable and not unduly influenced by any individual study. Despite the presence of publication bias, the overall conclusions of the study remained robust.

#### 3.3.2 Effects of adherence to ACSM guidelines on social interaction

This study conducted a systematic analysis of the effects of exercise interventions on social interaction in children with ASD. A total of 13 studies were included, of which 9 demonstrated high adherence to ACSM guidelines, and 4 were categorized as low adherence. Heterogeneity testing revealed *I*^2^ = 64%, indicating substantial heterogeneity (*I*^2^ > 50%); thus, a random-effects model was applied.

The standardized mean difference (SMD) in post-intervention social interaction scores between exercise and control groups was −0.22 (95% CI: [−0.54,0.99], *p* = 0.170), indicating a small, non-significant effect. Subgroup analyses revealed differential effects based on adherence to ACSM guidelines.

**Figure 6:**
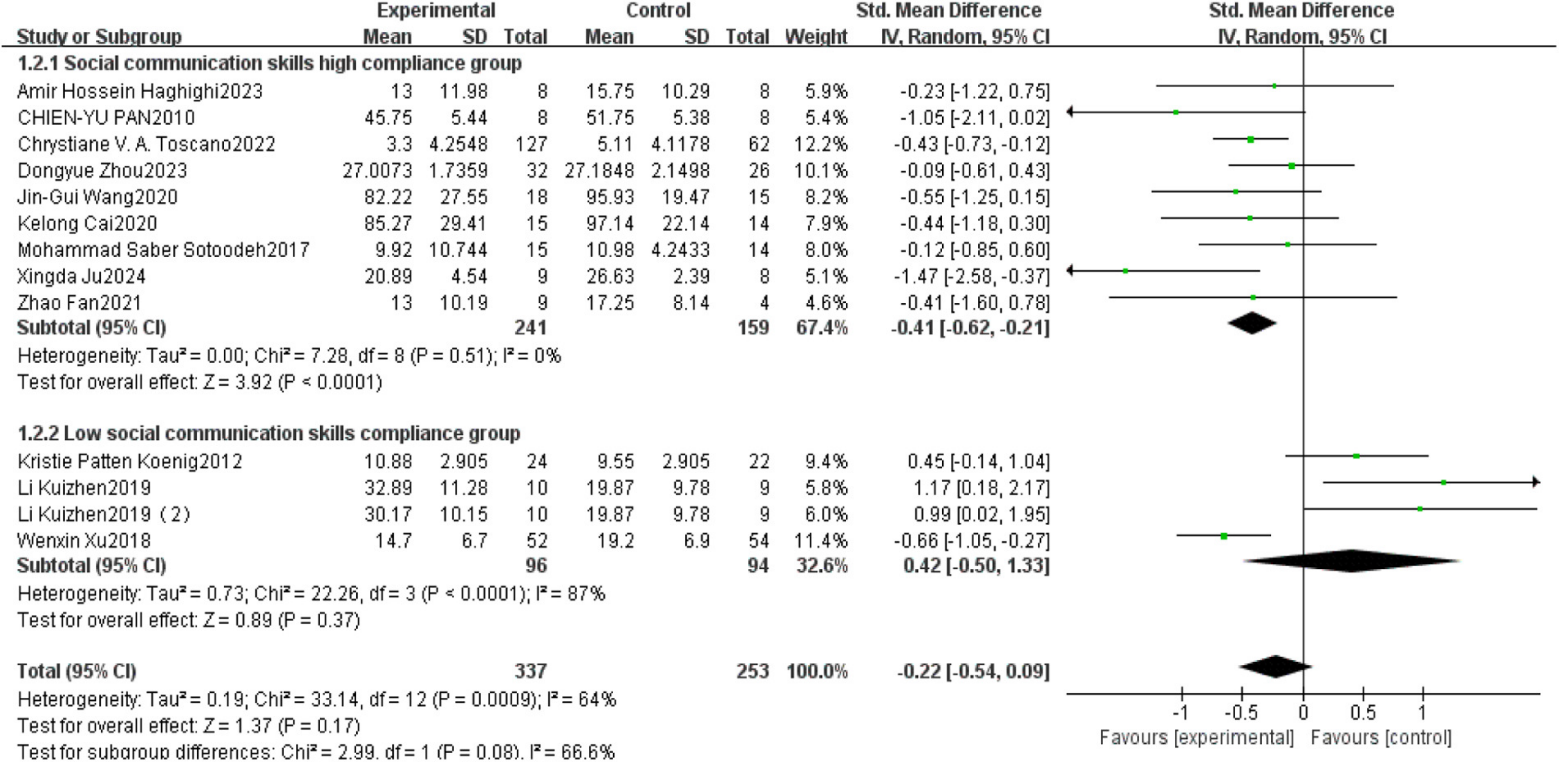
Forest plot of the impact of exercise intervention on the social interaction domain.

In the high adherence subgroup, the SMD was −0.41 (95% CI: [−0.62,−0.21], *p* < 0.001), with *I*^2^ = 0%. This suggests a statistically significant and reliable positive effect of exercise on social interaction, supported by the narrow confidence interval and absence of heterogeneity. Conversely, in the low adherence subgroup, the SMD was 0.42 (95% CI: [−0.50,1.33], *p* = 0.370), with high heterogeneity (*I*^2^ = 87%), indicating a non-significant effect with poor consistency across studies (Figure 6).

In summary, exercise interventions with high adherence to ACSM recommendations had a significant and robust effect on social interaction, whereas low adherence interventions showed no significant effect and high heterogeneity.

**Figure 7:**
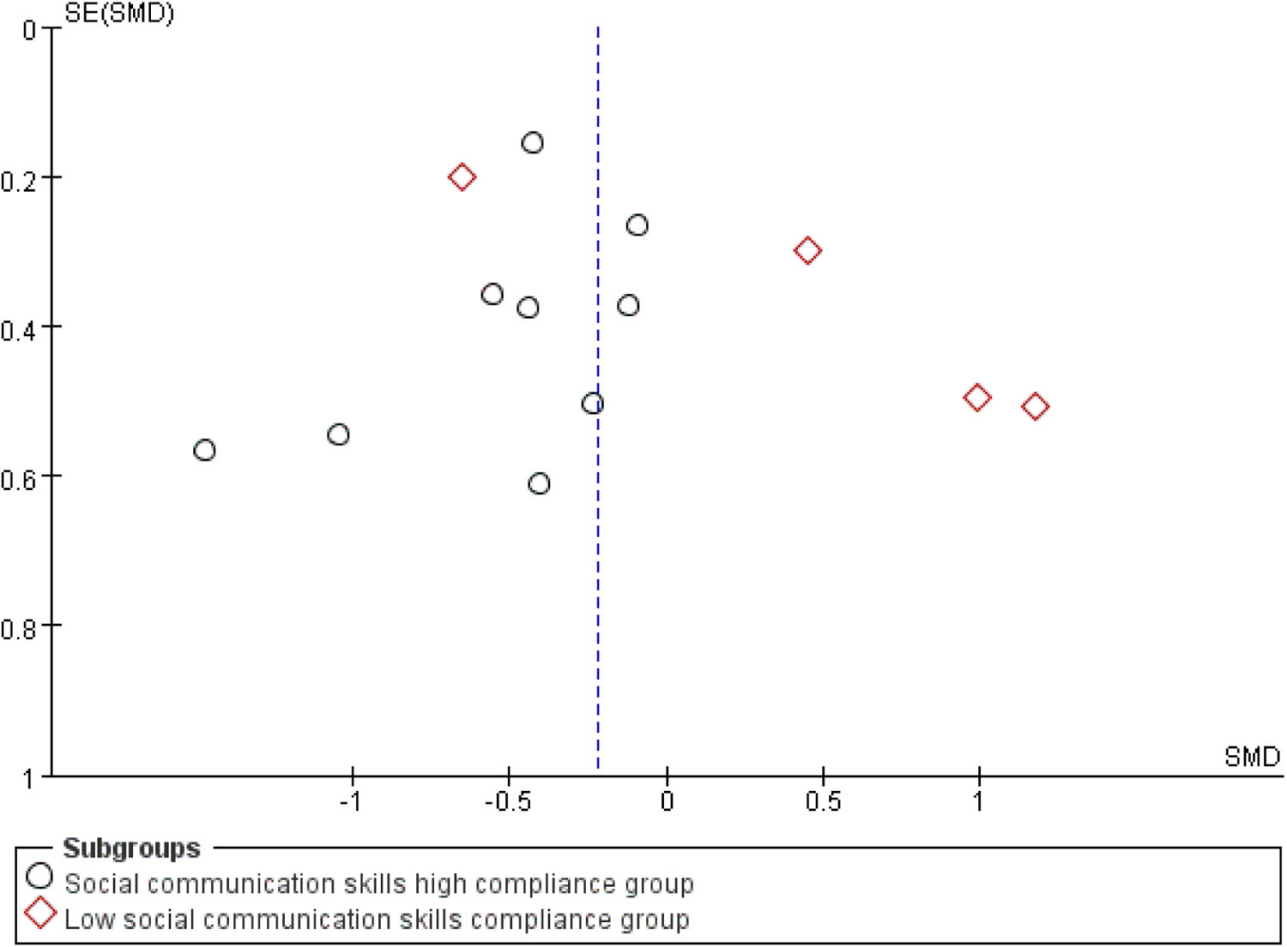
Funnel plot of the impact of exercise intervention on the social interaction domain.

Publication bias and sensitivity analyses were subsequently performed to validate the robustness of the findings. Visual inspection of the funnel plot (Figure 7) revealed near symmetry, indicating no apparent publication bias. Quantitative assessment using Begg's test (*p* = 0.903) and Egger's test (*p* = 0.483) further confirmed the absence of significant publication bias. This suggests that small-study effects or other potential biases had minimal influence on the results.

**Figure 8:**
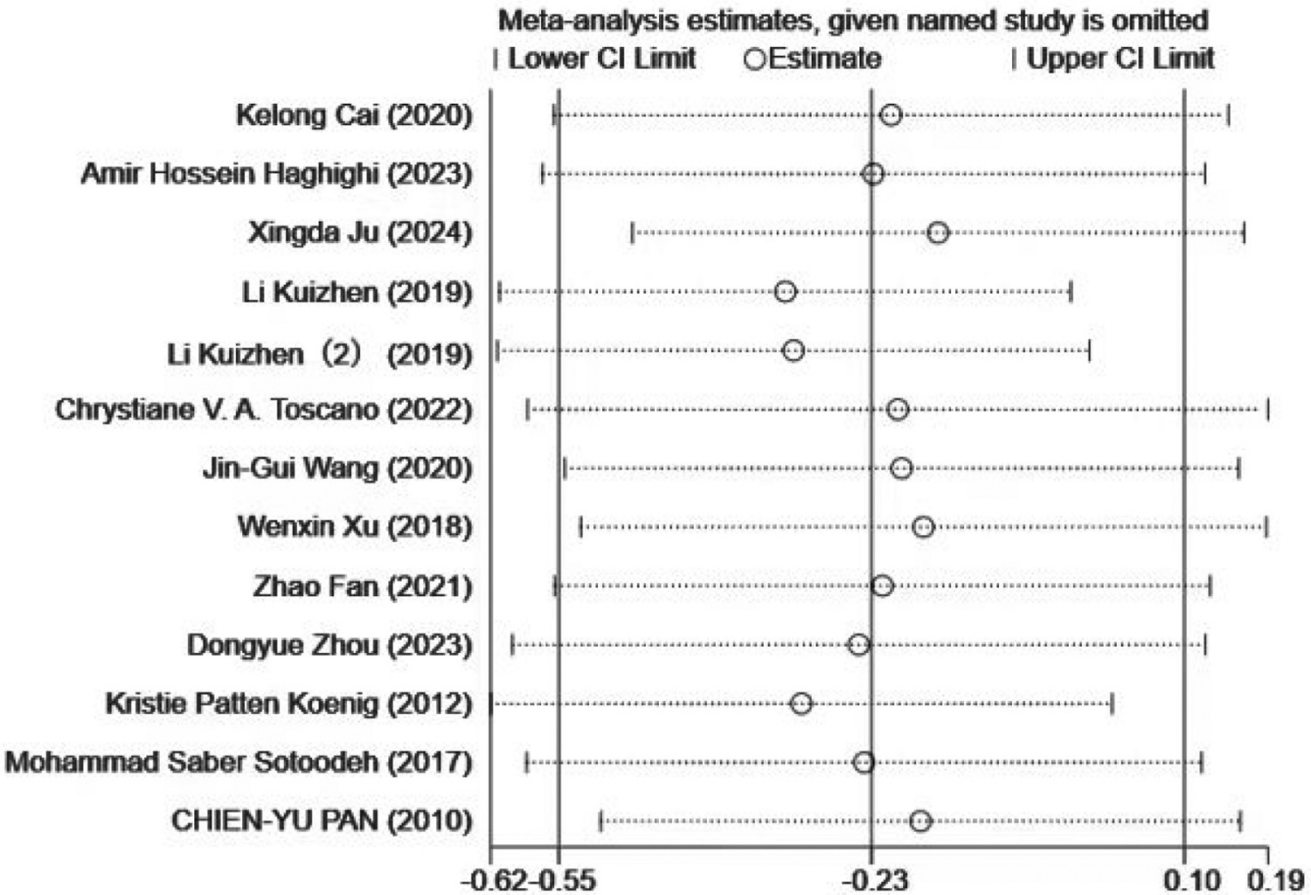
Sensitivity analysis of the impact of exercise intervention on social competence.

Sensitivity analysis (Figure 8) was conducted by sequentially removing each individual study, showing that no single study materially altered the overall effect estimate. This further supports the robustness of the findings, as the overall effect estimate remained stable even when individual studies were excluded.

#### 3.3.3 Effects of adherence to ACSM guidelines on behavioral patterns

This study conducted a systematic analysis of the effects of exercise interventions on behavioral patterns in children with ASD. A total of 11 studies were included, with 8 showing high adherence to ACSM guidelines and 3 categorized as low adherence. Heterogeneity testing revealed *I*^2^ = 79%, indicating substantial heterogeneity (*I*^2^ > 50%); therefore, a random-effects model was used for analysis.

The overall standardized mean difference (SMD) between the exercise and control groups for post-intervention behavioral scores was −0.79 (95% CI: [−1.26,−0.32], *p* = 0.001). These results indicate that exercise interventions had a significant positive effect on behavioral patterns in children with ASD, with a confidence interval that did not include zero.

Subgroup analysis further revealed the influence of adherence level on intervention outcomes. In the high adherence subgroup, the standardized mean difference (SMD) for post-intervention behavioral scores was −0.42(95% CI: [−0.73,−0.11], *p* = 0.008), with *I*^2^ = 47%. This indicates a statistically significant and reliable positive effect of exercise on behavioral patterns in children with ASD, supported by the narrow confidence interval and moderate heterogeneity.

**Figure 9:**
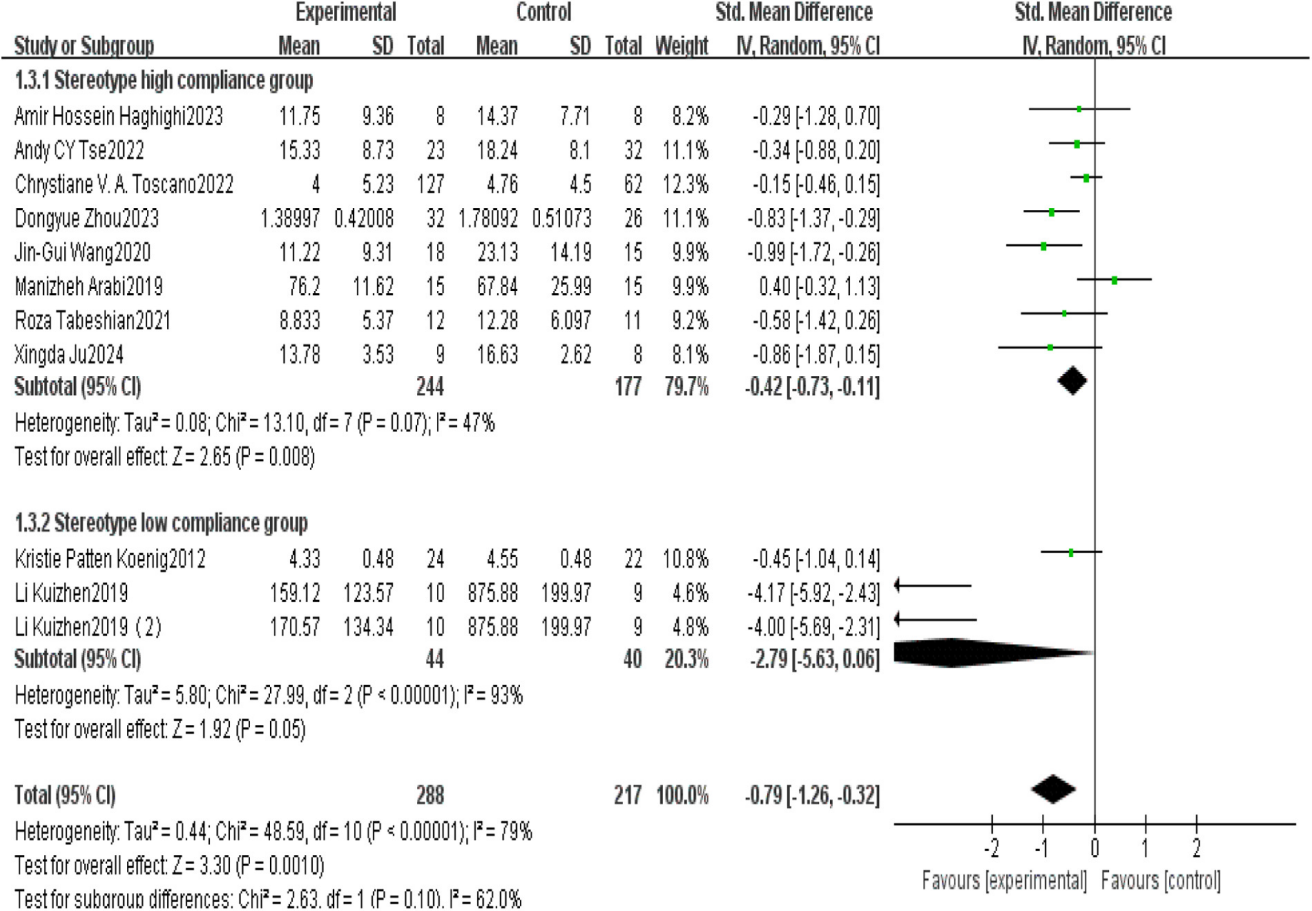
Forest plot of the impact of exercise intervention on behavioral pattern subdomains.

In the low adherence subgroup, the SMD was −2.79 (95% CI: [−5.63,0.06], *p* = 0.050), with *I*^2^ = 93%. This suggests that the effect was not statistically significant, as the confidence interval included zero and the extremely high heterogeneity (*I*^2^ = 93%) reflects low consistency across studies (Figure 9).

**Figure 10:**
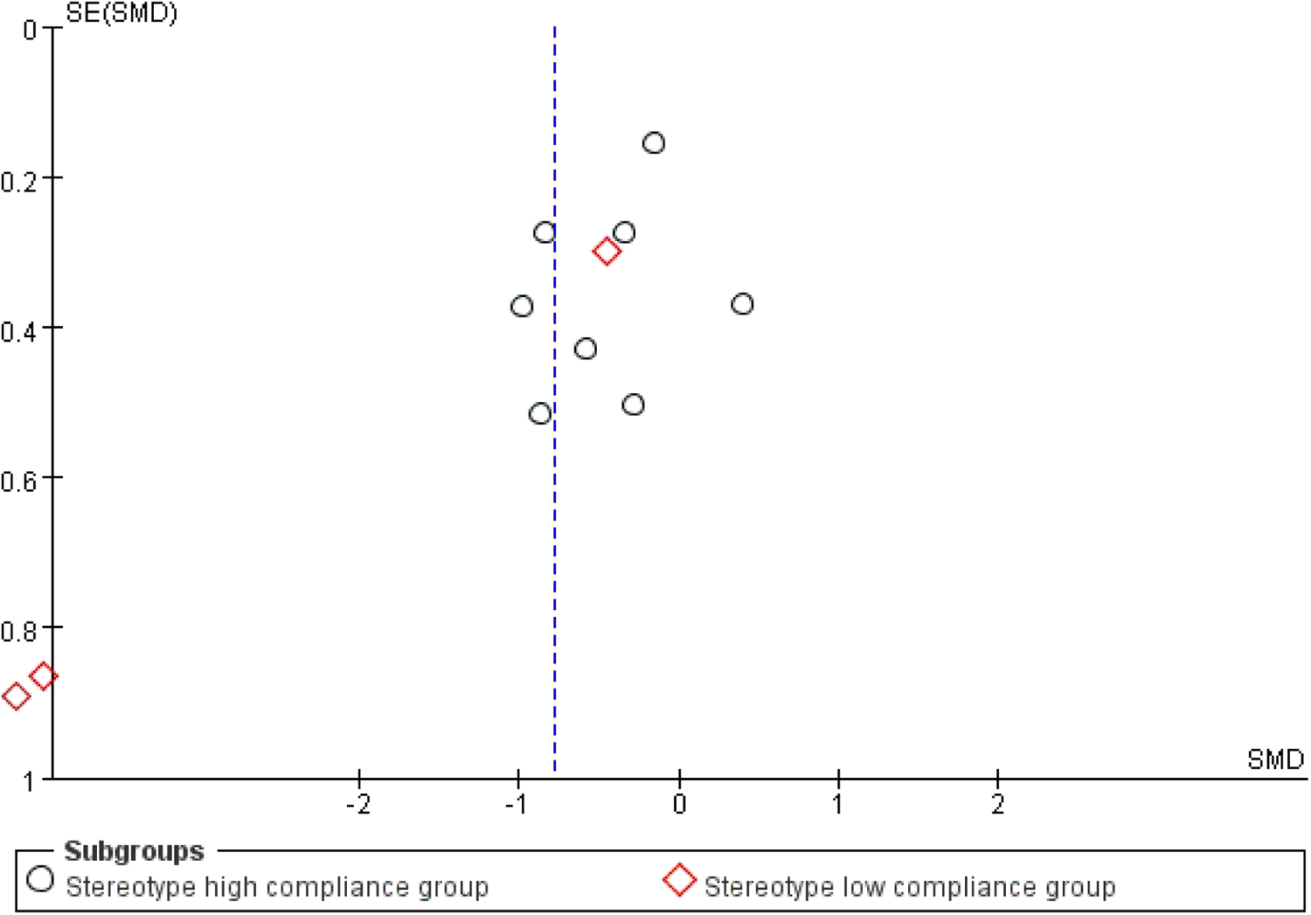
Funnel plot of the impact of exercise intervention on behavioral pattern domains.

Publication bias and sensitivity analyses were subsequently conducted for this outcome domain. Visual inspection of the funnel plot (Figure 10) showed near symmetry, initially suggesting the absence of substantial publication bias. However, statistical tests indicated otherwise: Begg's test (*p* = 0.016) and Egger's test (*p* = 0.015) both indicated the presence of significant publication bias. This suggests possible selective reporting, where studies with statistically significant results are more likely to be published, while those with null findings may be underreported.

**Figure 11:**
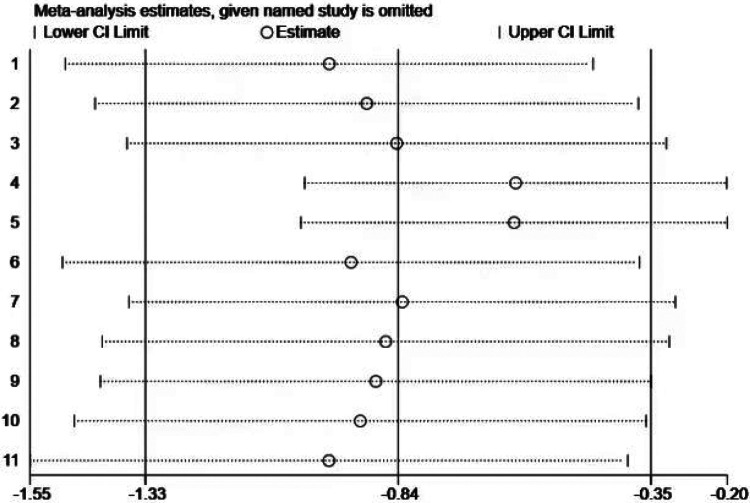
Sensitivity analysis of the impact of exercise intervention on behavioral pattern domains.

Sensitivity analysis (Figure 11) was conducted by sequentially removing each included study, and the results indicated that no single study had a material impact on the overall effect estimate. This finding suggests that, despite the presence of publication bias, the overall results remain stable and are likely to reflect the true effect.

However, due to the presence of publication bias, these results should still be interpreted with caution, as bias may introduce some distortion in the conclusions. Future research should further investigate the underlying causes of such bias and account for its potential impact when interpreting outcomes.

#### 3.3.4 Effects of adherence to ACSM guidelines on verbal and non-verbal communication

This study conducted a systematic analysis of language and nonverbal communication abilities in children with ASD to evaluate the effectiveness of exercise interventions. Ten studies were included, six of which demonstrated high adherence to the American College of Sports Medicine (ACSM) guidelines, while four exhibited low adherence. The heterogeneity test indicated substantial heterogeneity among studies (*I*^2^ = 91%, *I*^2^ > 50%), and therefore, a random-effects model was employed for statistical analysis. The overall analysis revealed that the difference in language and nonverbal communication scores between the exercise intervention group and the control group after completing clinical trials was not statistically significant (SMD = 0.33, 95% CI: [−0.31,0.91], *p* = 0.320), as the confidence interval included zero.

Further subgroup analyses indicated the influence of adherence level on intervention outcomes. In the high-adherence subgroup, the difference in language and nonverbal communication scores between the intervention and control groups was SMD = 0.21 (95% CI: [−0.14,0.57], *p* = 0.240), with an *I*^2^ of 45%. The confidence interval included zero, indicating no statistically significant effect. In the low-adherence subgroup, the difference was SMD = 0.59 (95% CI: [−1.67,2.84], *p* = 0.610), with an *I*^2^ of 98%, suggesting poor consistency among the included studies and no significant effect of the intervention.

**Figure 12:**
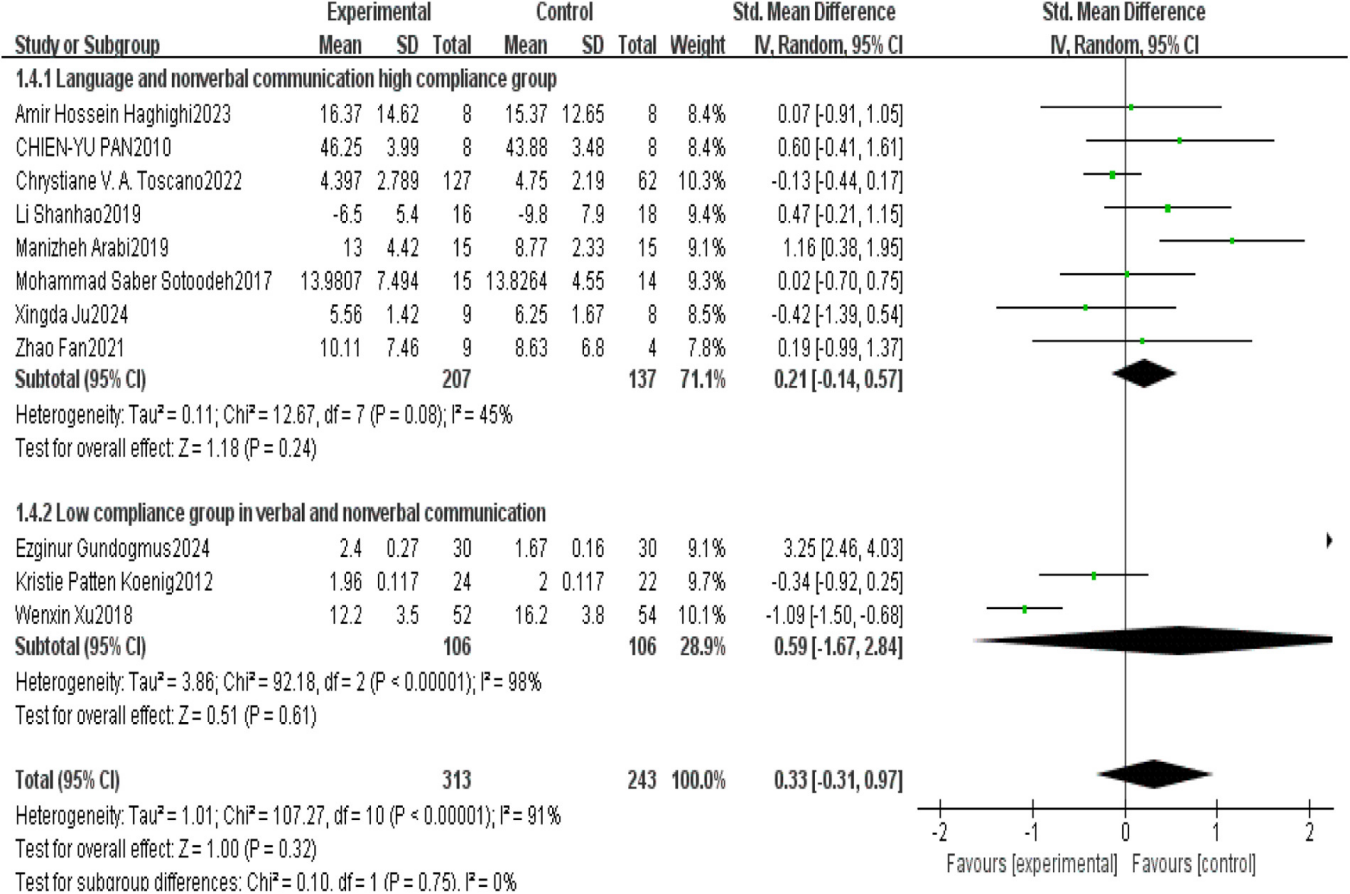
Forest plot of the effects of exercise intervention on verbal and non-verbal communication.

In summary, regardless of adherence to ACSM-recommended exercise protocols, no significant effects of exercise interventions on the language and nonverbal communication abilities of children with ASD were observed. These findings suggest that exercise interventions may not be an effective strategy for improving communication in children with ASD, or that optimization of intervention protocols may be necessary to enhance their efficacy (Figure 12).

**Figure 13:**
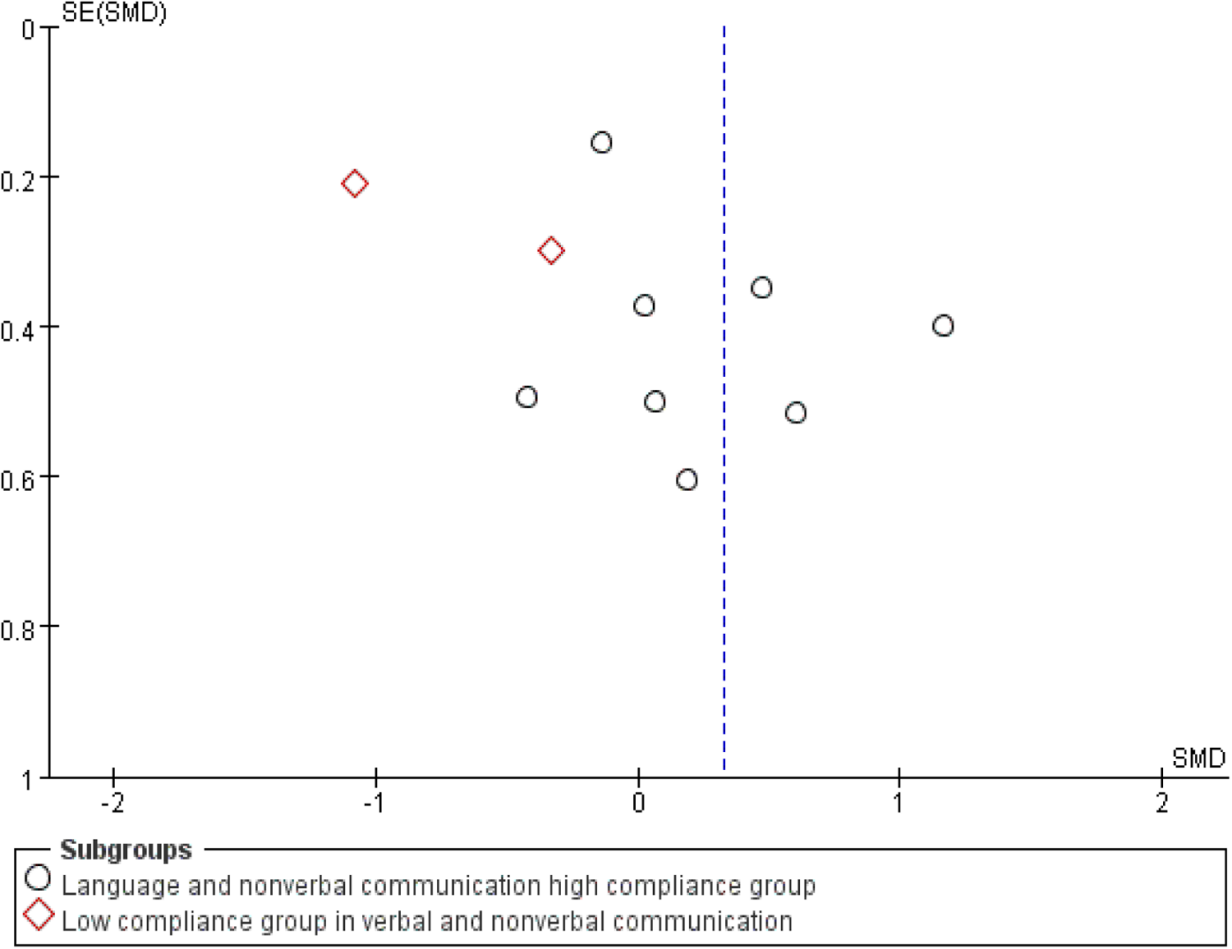
Funnel plot of the effects of exercise intervention on verbal and non-verbal communication.

This study further visually assessed potential publication bias using a funnel plot (Figure 13). The plot appeared approximately symmetrical on both sides, suggesting the absence of apparent publication bias. Subsequent statistical tests, including Begg's test (*p* = 0.102) and Egger's test (*p* = 0.17), revealed no significant publication bias, further supporting the robustness of the study findings.

**Figure 14:**
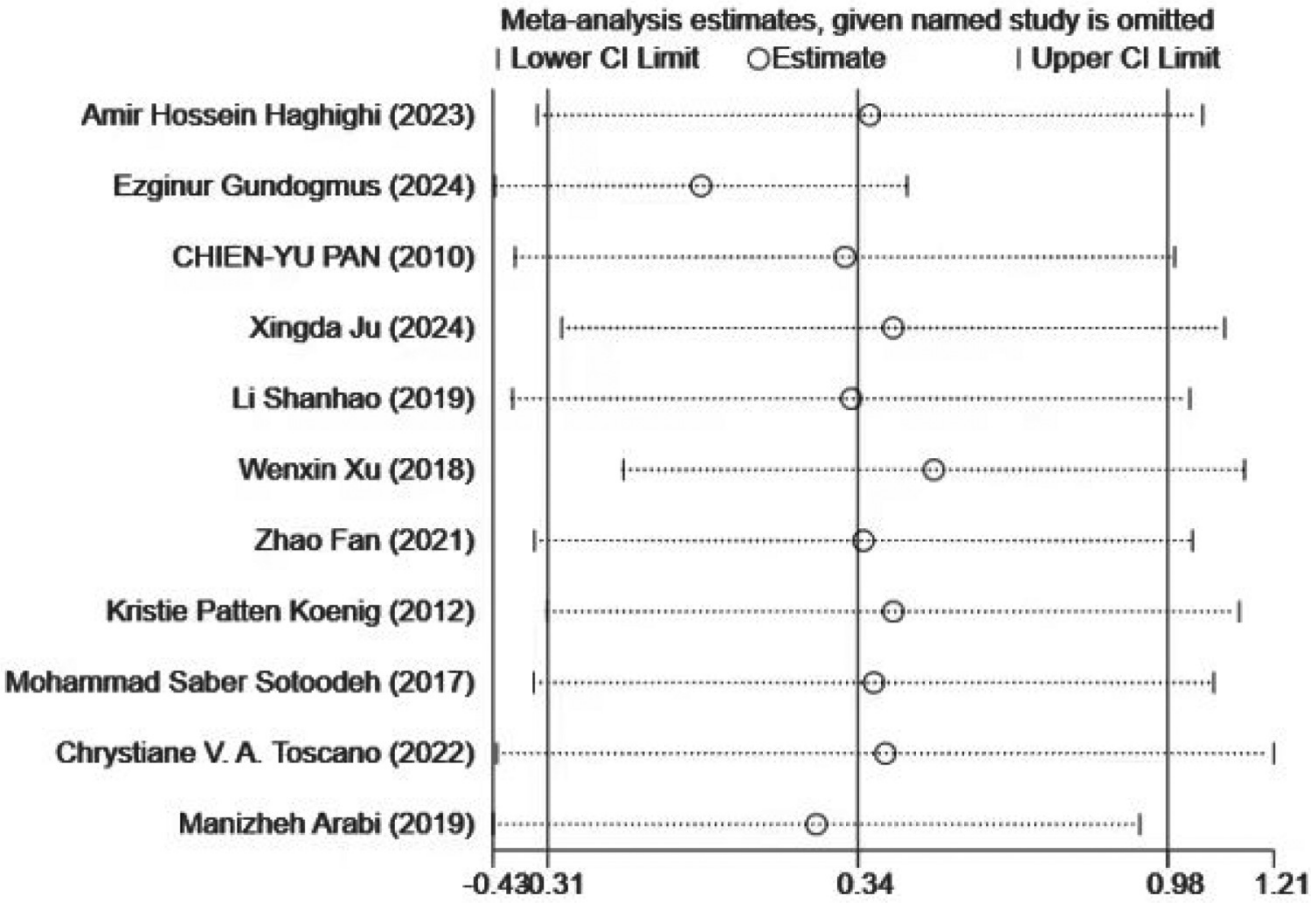
Sensitivity Analysis Diagram of the Impact of Exercise Intervention on Verbal and Nonverbal Communication.

In the sensitivity analysis (Figure 14), the sequential exclusion of each individual study indicated that no single study had a substantial impact on the overall results. This finding suggests that the results are robust, as the overall effect estimate remained stable even when individual studies were removed.

#### 3.3.5 Meta-regression analysis

Given the high level of observed heterogeneity, a meta-regression analysis was conducted to explore potential study-level characteristics that may have contributed to this variation. Participant characteristics, including health status, sex, and age, were initially considered. Studies that included participants with comorbid conditions were excluded; thus, health status was not incorporated as a factor in the meta-regression. Regarding sex, among studies that reported gender distribution, the combined male-to-female ratio was 575:139, which is consistent with the known prevalence rate of autism (approximately 3–4:1). Due to this significant sex imbalance, sex was not included as a factor in the meta-regression analysis. With respect to age, all participants were children, and age differences across studies were minimal. Furthermore, some studies did not report participant age, posing challenges to its inclusion; thus, age was excluded as a covariate.

Therefore, sample size and year of publication were included as potential sources of heterogeneity in the meta-regression analysis. The results of the meta-regression analyses across various outcome domains—including motor skills, social interaction domains, behavioral pattern domains, and language and nonverbal communication domains—revealed no statistically significant differences after adjusting for covariates such as sample size, year of publication, and overall composite factors (Table 8).

**Table 8:** Meta-regression analysis of the impact of different study characteristics on inter-study heterogeneity.

Result	Variable	Regression coefficient	Standard deviation	Z	*P* > |Z|	95%
Motor skills group	Year of Publication	0.1341697	0.219994	0.61	0.542	[−0.2970106,0.5653499]
	Sample size	0.1558305	0.986084	1.58	0.114	[−0.0374383,0.3490994]
Social Interaction Ability Group	Publication Year	−0.0218528	0.0523204	−0.42	0.676	[−0.124399,0.0806933]
	Sample size	−0.0016323	0.003816	−0.43	0.669	[−0.009115,0.0058469]
Behavioral pattern functional block	Publication Year	0.037173	0.1372893	0.27	0.787	[−0.2319091,0.3062551]
	Sample size	0.0082691	0.0089548	0.92	0.356	[−0.0092819,0.258201]
Verbal and Nonverbal Ability Groupings	Publication Year	0.0723613	0.085206	0.85	0.396	[−0.0946393,0.2393619]
	Sample size	−0.004807	0.0070939	−0.68	0.498	[−0.0187108,0.0090968]

## 4 Discussion

This systematic review and meta-analysis investigated the impact of high versus low adherence to the American College of Sports Medicine (ACSM) recommendations. The analysis was based on data from 27 studies encompassing 29 exercise interventions, including 18 studies with high adherence and 11 with low adherence to the ACSM guidelines.

### 4.1 Positive impact of exercise interventions on children with ASD

Previous research has indicated that the effects of exercise interventions on social interaction, language, and nonverbal communication remain inconclusive. However, interventions with high adherence to the American College of Sports Medicine (ACSM) guidelines demonstrated significant improvements in social interaction, suggesting that exercise type, frequency, and intensity may play a critical role in therapeutic outcomes. Our findings further support this view, showing that a higher level of exercise dosage adherence—aligned with ACSM recommendations—was more beneficial in alleviating symptoms among children with ASD compared to interventions with lower adherence.

### 4.2 Adherence to exercise according to ACSM guidelines

One of the key strengths of this study is its integration of various exercise modalities, intensities, durations, and other relevant indicators employed in prior research. The study utilized adherence to the American College of Sports Medicine (ACSM) guidelines as a grouping variable to evaluate the effect of exercise dosage on symptom improvement in children with ASD. A central aspect of this study lies in the definition and operationalization of ACSM adherence, which provided a standardized framework for comparison across interventions. Based on this framework, we conducted a focused analysis specifically examining the influence of ACSM adherence across different exercise types on ASD symptomatology. The research design included analyses of aerobic exercise, resistance training, and stretching interventions.

### 4.3 The impact of ACSM adherence on the effectiveness of exercise interventions

In this study, we aimed to include as many types of exercise interventions as possible to assess the impact of adherence to the American College of Sports Medicine (ACSM) guidelines on intervention effectiveness. A meta-analysis with subgroup analyses based on adherence to ACSM-recommended exercise dosage revealed that interventions with high ACSM adherence were more effective than those with low adherence in improving motor skills, social interaction, and behavioral patterns in children with ASD.

For the social interaction domain, the high-adherence group showed a statistically significant effect (SMD = −0.41, 95% CI: [−0.62,−0.21]), whereas the low-adherence group did not (SMD = −0.42, 95% CI: [−0.5,1.33]). In the behavioral pattern domain, the high-adherence group also demonstrated a significant improvement (SMD = −0.35, 95% CI: [−0.55,−0.15]), while the low-adherence group did not (SMD = −2.79, 95% CI: [−5.63,0.06]). These findings suggest that high adherence to ACSM-guided exercise significantly benefits social interaction and behavioral outcomes in children with ASD.

When examining the impact of ACSM adherence on motor skills, the improvement was greater in the high-adherence group (SMD = 1.44) than in the low-adherence group (SMD = 1.26), and the narrower confidence interval in the high-adherence group indicates greater reliability of the results. In contrast, exercise interventions based on ACSM guidelines had limited effects on language and nonverbal communication, with neither subgroup demonstrating statistical significance.

In conclusion, consistent with multiple studies, adherence to ACSM-recommended exercise regimens appears to have a positive influence on various symptoms in children with ASD.

### 4.4 Other influencing factors

This study exhibited a degree of heterogeneity, which was addressed using sensitivity and subgroup analyses. The results remained robust following the sequential exclusion of individual studies. Similar to pharmacological treatment, exercise therapy has been explored as an alternative method for managing symptoms in children with ASD. In the process of determining optimal exercise dosage, it is essential to provide a detailed description of the intervention protocol to establish an appropriate dosage range. However, the variability across studies has increased the complexity of identifying optimal exercise modalities and dosage parameters.

Based on current evidence, we can only infer that physical activity may improve symptoms in children with ASD; no specific exercise regimen has been proven to be superior to others. Current research remains focused on comparing the effects of specific aspects of exercise interventions, and there is a lack of standardized guidelines for determining optimal exercise protocols and dosages. Although the American College of Sports Medicine (ACSM) provides general exercise recommendations, there remains controversy and uncertainty regarding their application to ASD populations. Nonetheless, our findings support the feasibility of implementing ACSM-recommended exercise prescriptions in this population.

### 4.5 Limitations and future directions of this study

Despite the valuable findings and contributions of this study, several limitations should be acknowledged. First, the study relied on meta-analyses of previously published research, making it inherently subject to the limitations and potential biases present in the original studies. Another limitation is the absence of standardized protocols for exercise interventions and dosage across the included studies. The variability in exercise type, intensity, duration, and frequency poses a challenge to drawing definitive conclusions regarding the optimal exercise dosage for improving symptoms in children with ASD.

Future research should focus on developing standardized intervention protocols and guidelines tailored to this population. Looking ahead, well-designed, large-scale randomized controlled trials (RCTs) with extended follow-up periods are essential. These studies should incorporate standardized exercise protocols and consider individual characteristics—such as age, sex, and comorbidities—to enable the effective personalization of exercise interventions.

## 5 Conclusion

Exercise interventions demonstrate positive effects on motor skills and behavioral patterns in children with ASD. In exploring optimal exercise dosage, findings indicate that interventions with high adherence to the American College of Sports Medicine (ACSM) guidelines are more effective than low-adherence interventions in improving motor skills, social interaction, and behavioral patterns in children with ASD, with particularly pronounced effects observed in the domains of social interaction and behavioral patterns.

## Data Availability

The datasets presented in this study can be found in online repositories. The names of the repository/repositories and accession number(s) can be found in the article/Supplementary Material.
