# Supplementary material for: Effects of exercise dosage on children with autism spectrum disorder: a systematic review and meta-analysis of randomized controlled trials
Source: Front Child Adolesc Psychiatry. 2025 Sep 4;4:1647280. doi: 10.3389/frcha.2025.1647280 (PMC12444209; doi:10.3389/frcha.2025.1647280)
Supplement: Supplementary file 1 [file Datasheet1.doc]

| Search | PUBMED |
| --- | --- |
| #1 | Search: ****(autism[MeSH Terms]) OR (Autism Spectrum Disorder[MeSH Terms])**** |
| #2 | Search: ****((((Pervasive Developmental Disorders[Title/Abstract]) OR (autism[Title/Abstract])) OR (Autism Spectrum Disorder[Title/Abstract])) OR (Asperger[Title/Abstract])) OR (PDD-NOS[Title/Abstract])**** |
| #3 | Search: ****(#1) OR (#2)**** |
| #4 | Search: ****Exercise[MeSH Terms]**** |
| #5 | | Search: ****(((((((((((((Exercise[Title/Abstract]) OR (Exercises[Title/Abstract])) OR (Sports[Title/Abstract])) OR (Physical Activity[Title/Abstract])) OR (Motor Activity[Title/Abstract])) OR (Training[Title/Abstract])) OR (endurance training[Title/Abstract])) OR (Tai Chi[Title/Abstract])) OR (yoga[Title/Abstract])) OR (Balance[Title/Abstract])) OR (Resistance[Title/Abstract])) OR (Flexibility[Title/Abstract])) OR (Cardiovascular[Title/Abstract])) OR (Aerobic[Title/Abstract])**** | | --- | |
| #6 | Search: ****(#4) OR (#5)**** |
| #7 | Search: ****(#3) AND (#6)**** |

| search | Web science |
| --- | --- |
| #1 | Search:(((((((((((((TI=(Exercise))OR TI=(Exercises)) OR TI=(Sports)) OR TI=(Physical Activity)) OR TI=( Motor Activity)) OR TI=(Training )) OR TI=(endurance training)) OR TI=(Tai Chi)) OR TI=(yoga)) OR TI=(Balance)) OR TI=(Resistance)) OR TI=(Flexibility)) OR TI=(Cardiovascular)) OR TI=(Aerobic) and Preprint Citation Index (Exclude – Database) |
| #2 | ((((TI=(Pervasive Developmental Disorders )) OR TI=(autism )) OR TI=(Autism Spectrum Disorder)) OR TI=(Asperger)) OR TI=(PDD-NOS) and Preprint Citation Index (Exclude – Database) |
| #3 | #1 AND #2 |

| search | EMBASE |
| --- | --- |
| #1 | exercise:ti OR exercises:ti OR sports:ti OR 'physical activity':ti OR 'motor activity':ti OR training:ti OR 'endurance training':ti OR 'tai chi':ti OR yoga:ti OR balance:ti OR resistance:ti OR flexibility:ti OR cardiovascular:ti OR aerobic:ti |
| #2 | 'pervasive developmental disorders':ti OR autism:ti OR 'autism spectrum disorder':ti OR asperger:ti OR 'pdd nos':ti |
| #3 | #1 AND #2 |

| search | Cochrane |
| --- | --- |
| #1 | (Pervasive Developmental Disorders):ti,ab,kw OR (autism):ti,ab,kw OR (Autism Spectrum Disorder):ti,ab,kw OR (Asperger):ti,ab,kw OR (PDD-NOS):ti,ab,kw |
| #2 | (Exercise):ti,ab,kw OR (Exercises):ti,ab,kw OR (Sports):ti,ab,kw OR (Physical Activity):ti,ab,kw OR (Motor Activity):ti,ab,kw |
| #3 | (Training):ti,ab,kw OR (endurance training):ti,ab,kw OR (Tai Chi):ti,ab,kw OR (yoga):ti,ab,kw OR (Balance):ti,ab,kw |
| #4 | (Training):ti,ab,kw OR (endurance training):ti,ab,kw OR (Tai Chi):ti,ab,kw OR (yoga):ti,ab,kw OR (Balance):ti,ab,kw |
| #5 | (#2 OR #3 OR #4) |
| #6 | #1 AND #5 |
